# Pediatric Emergency Medicine Disaster Simulation Curriculum: The 5-Minute Trauma Assessment for Pediatric Residents (TRAP-5)

**DOI:** 10.15766/mep_2374-8265.10940

**Published:** 2020-08-21

**Authors:** Tavis Dickerson-Young, Ashley Keilman, Hiromi Yoshida, Maya Jones, Nathan Cross, Anita Thomas

**Affiliations:** 1 Fellow, Pediatric Emergency Medicine, University of Washington; 2 Assistant Professor, Department of Pediatrics, University of Washington; 3 Assistant Professor, Department of Radiology, University of Washington

**Keywords:** Trauma, Pediatric Trauma, Pediatric Emergency Medicine, Emergency Medicine, Primary Survey, Disaster, Mass Casualty, Pediatrics, Simulation

## Abstract

**Introduction:**

Pediatric trauma management is a high-stress, high-risk, low-frequency event, and exposure through simulation can help identify and address knowledge gaps. Pediatric residents are likely to provide care for children with traumatic injuries, and it is important they are skilled in performing a rapid trauma assessment.

**Methods:**

We developed a simulation-based rapid pediatric trauma assessment curriculum for pediatric residents in the setting of a mass casualty disaster. The patients were 5-year-olds portrayed by mannequins with varying injuries including intracranial hemorrhage, solid organ injury, and open extremity fractures. Critical actions included assigning roles, completing primary assessment within 2 minutes, and giving summary statement and management priorities within 5 minutes using clear communication techniques. We created a badge-sized reference card as well as scenario-specific debriefing tools to facilitate assessment and discussion of learning objectives following the simulation.

**Results:**

We conducted two sessions with a total of 49 participants. The case was rated as highly relevant (session 1, *m* = 4.7; session 2, *m* = 4.9) and realistic (session 1, *m* = 4.8; session 2, *m* = 4.4) by participants on a 5-point Likert scale. During the two sessions participants completed the primary survey in an average of 2.46 and 2.29 minutes, respectively, and the secondary survey with summary statement in an average of 5.08 and 4.27 minutes, respectively.

**Discussion:**

This educational resource supports the setup, production, and debriefing of a low-fidelity simulation focused on the pediatric trauma assessment for the novice learner. Also included are educational reference materials and a participant evaluation form.

## Educational Objectives

By the end of this activity, learners will be able to:

1. Demonstrate the ability to successfully complete the primary survey for pediatric trauma patients within 2 minutes of patient arrival.
2. Verbalize and prioritize the next two steps in pediatric trauma management within 5 minutes of patient arrival.
3. Assign and maintain team roles.
4. Demonstrate effective teamwork and communication.

## Introduction

The majority of pediatric trauma is the result of blunt injury, which frequently causes multisystem trauma.^1^ Potential injuries are vast and can include life-threatening airway obstruction, inadequate breathing, abdominal/pelvic hemorrhage, and intracranial hemorrhage among others.^1^ The primary survey is a structured method to rapidly assess and intervene on immediate life-threatening conditions.^1^ After initial assessment and stabilization during the primary survey, subsequent actions include completing a secondary survey, conducting a rapid surgical consultation, and obtaining radiologic or laboratory studies to assess the extent and severity of injury. As the goal of the primary survey is to identify imminently life-threatening conditions, failure to complete the primary survey in a succinct and efficient manner is associated with worse patient outcomes.^1,2^ Pediatric trauma is a relatively low-frequency, but high-stress and high-risk occurrence, particularly in a mass casualty incident. Thus, novice learners or clinical staff without regular exposure to pediatric trauma may fail to rapidly and effectively complete the trauma assessment.^2^ Given that this is a high-risk, but low-frequency event, simulation provides an alternative method to practice these skills.

According to the Centers for Disease Control and Prevention (CDC), unintentional injuries are the leading cause of death for children and adolescents from one to 24 years of age in the United States.^3^ This represents a significant number of children with trauma that will require medical care each year, and these patients are likely to receive care at a local emergency room or trauma center, whose staff should be trained for pediatric trauma.^4^ The most common form of pediatric trauma across all age groups is motor vehicle crashes, with other leading etiologies including assault, falls, burns, and sports injuries.^5^ Mass casualty incidents are rare and represent only approximately 0.2% of emergency medical services (EMS) responses according to a national EMS database.^6^ However, mass casualty events are commonly associated with response delays and have the potential to overwhelm receiving centers.^6^ Patients not transported by EMS frequently present to the closest emergency room, which is not necessarily a designated trauma center, so it is important that staff who could potentially care for children be prepared to efficiently and effectively assess the pediatric trauma patient.

The content of this simulation scenario is targeted at novice to intermediate learners of pediatric trauma, such as pediatric residents. Residents and other trainees are likely to be involved in the care of children with traumatic injuries, so it is important that they be skilled and equipped to perform a rapid trauma assessment. Simulated exposure to pediatric trauma can help identify and address knowledge gaps, particularly with pediatric residents who may not always be the primary responders. In the setting of a mass casualty or disaster, pediatric trainees may be heavily utilized. While these incidents are relatively rare, they are high-risk. An interactive, hands-on scenario with timed and focused assessment may strengthen and solidify knowledge of pediatric trauma. While there are cases available through *MedEdPORTAL* that simulate pediatric trauma,^7–10^ there are currently no published resources that include a timed pediatric primary survey assessment or have teams of learners simultaneously assess multiple pediatric casualties in the setting of a disaster. This curriculum may be used independently or in a series with simulation cases from the Pediatric Emergency Medicine Simulation Curriculum.

## Methods

### Development

Pediatric Emergency Medicine physicians with experience in curriculum development and simulation developed this simulation as part of a pediatric residency simulation curriculum for formative learning. The scenarios were created to represent the types of child injuries that may present during a mass casualty event. Through participation in this scenario, participants were expected to complete a primary trauma survey within 2 minutes, identify critical management interventions, and state their next two management priorities of a pediatric trauma patient. There was no specific prerequisite knowledge required other than being a health care provider that cares for acutely ill pediatric patients. However, it may be helpful for participants to have a basic understanding of Advanced Trauma Life Support (ATLS) algorithms. As the curriculum was developed to target pediatric trainees who generally were not ATLS trained, we created a brief educational presentation that was delivered prior to the simulation (Appendix F) to prebrief participants on the simulation.

### Equipment/Environment

This scenario was conducted in a conference room with mobile simulation equipment and low-fidelity mannequins; it may also be conducted in an emergency department (ED) or a simulation lab functioning as an ED. Low-fidelity child mannequins were used for the scenarios (Appendix A). The cases could be modified to younger and/or older children depending on mannequin availability. Medications and equipment typically available in ED trauma management were made available to participants (Appendix B). The facilitator asked participants to group themselves into four teams to evaluate patients upon arrival and explained that the patients would arrive on stretchers in 3 minutes. No parents were available for additional history. Facilitators or embedded participants functioned as bedside nurses for obtaining IV access, drawing labs, and administering medications, as well as facilitated the simulation and provided management suggestions if the team struggled. If additional facilitators are available, they can play the role of an adult teacher who presents with the children and reports that they were previously healthy.

We utilized noon educational time to conduct this simulation and had four scenarios running simultaneously (i.e., two stations with Patient A and two stations with Patient B). The ideal team size was five to six trainees per patient. During the first session, participants were told that a school bus containing a kindergarten class was involved in a collision and overturned on the street in front of the hospital. There were multiple patients who all required a full trauma assessment. For the second session, participants were told that the gymnasium roof of an elementary school near the hospital had collapsed. Although the simulation medical content remained constant, the type of disaster was changed between the sessions so that participants would not anticipate patients having the same injuries. The type of disaster could be adjusted based on risks specific to your region (tornado, earthquake, hurricane, fire, etc.). The scenario was designed for a minimum of two patients, Patient A and Patient B, but facilitators may increase the number of patients depending on the number of participants (two or more patients representing Patient A and two or more patients representing Patient B).

#### Patient A

This male patient was found moaning and with eyes closed by medics next to the overturned school bus and transported to the ED on a stretcher. The team encountered the patient initially laying on a backboard in bed with eyes open, crying, and bleeding from his right upper arm. Blood soaked gauze obscured a bleeding extremity with visible deformity. There was a four cm boggy occipital hematoma without scalp bleeding. There was no IV access, and he was not yet on monitors.

#### Patient B

This female patient was found moaning and with eyes closed by medics next to the overturned school bus and transported to the ED on a stretcher. The team encountered the patient initially laying on a backboard in bed with eyes open, crying, and bleeding from her left thigh. Blood soaked gauze obscured a bleeding extremity with visible deformity. There was a large abdominal bruise in the left upper quadrant. There was no IV access and she was not yet on monitors.

Throughout the scenario, the simulation facilitator provided clinical updates as the learners progressed through the case and point of care laboratory findings upon request. Electronic or paper copies of X-rays were provided upon request, and advanced imaging was reviewed after the scenario if time permitted (Appendix C and Appendix F).

### Personnel

Each simulation session was attended by approximately 25 pediatric residents, and attendance was optional. The two sessions were performed at the same site but were separated temporally such that all residents had new clinical duties and team assignments at the time of the second session. This strategy was chosen to maximize the number of unique participants. Because surveys were anonymous, the number of unique participants could not be determined, although it was thought that the majority of surveys filled out were unique. If participants were present at both sessions, they were assigned to a different patient if possible. By design, all roles were filled by physicians; however, the simulation could be run in an interdisciplinary setting with attending and trainee physicians, nurses, respiratory therapists, and other clinical staff who would respond to a mass casualty event. If members of other disciplines are present, they should function in their usual clinical role. Four facilitators with experience in Pediatric Emergency Medicine and expertise in facilitation/debriefing methods conducted this simulation, with one facilitator for each patient. Participants had prior experience with simulation, and many had previous experience with pediatric trauma resuscitation.

### Implementation

The simulation sessions occurred during resident noon conference and lasted 45 minutes. Twenty-five minutes were devoted to didactic lecture material, 5 minutes were devoted to orientation to the activity, 5 minutes were devoted to participation in the timed simulation, and 10 minutes were devoted to debriefing. Debriefing was conducted in the same space, but a separate space could be used if available. To begin, the participants were informed of the mass casualty and that multiple victims were being transported to the ED by medics. Each patient needed a full trauma assessment, and participants were instructed to divide into four groups to simultaneously assess the first four patients that would be arriving in 3 minutes (two patients representing Patient A and two patients representing Patient B). The scenario was designed for multiple patients to be assessed simultaneously to better simulate mass casualty. Patient A had an exam notable for a four cm boggy occipital hematoma without scalp bleeding, a Glascow Coma Scale of 14, possible cervical spine tenderness, right upper arm deformity with active bleeding, and a decreased right radial pulse (see Appendix A for full exam). Patient B had an exam notable for left thigh deformity with active bleeding and a decreased left dorsalis pedis pulse, as well as a large bruise on the abdomen in the left upper quadrant with tenderness to palpation and guarding, and a Glascow Coma Scale of 14 (see Appendix A for full exam). Participants were expected to assign team roles and complete the primary survey within 2 minutes, and then complete the secondary survey and verbalize the next two priorities in management within 5 minutes of beginning the simulation. These time limits and expectations were restated just prior to beginning the simulation. Facilitators used stopwatches to monitor and record time. The case concluded after five minutes or when participants completed their secondary survey and verbalized their next two priorities in management. If participants had not completed these steps at the 5-minute mark, the facilitator asked the team leader for a summary and the team's next two priorities. If time permits for a longer session (60–75 minutes), participants could then be asked to assess the next patient arriving from the mass casualty event by switching patients with another group (group with Patient A moves to Patient B and vice versa).

Facilitation of the scenario and debriefing was led by pediatric emergency medicine physicians with training in simulation. Simulation technicians used Appendix A to plan and prepare low-fidelity pediatric mannequins. Appendix B provides a list of recommended equipment and medications commonly found in the ED environment. Participants were given the primary survey badge reference card in Appendix C following the didactic for use during the scenario and in clinical use beyond this session. Imaging and lab results for use during the case and the debrief are also included in Appendix C. Images of both subdural and epidural hemorrhage are provided. Facilitators may choose which intracranial injury to apply to Patient A as they prefer, or they may use the images to facilitate discussion of injury patterns and potential imaging findings during the debrief. Similarly, images of both liver and splenic lacerations are provided. Facilitators may choose which solid organ injury to apply to Patient B as they prefer, or they may use the images to facilitate discussion of injury patterns and potential imaging findings during the debrief. Facilitators were provided with the glossary of teamwork and communication tools in Appendix D to review prior to the session in order to help establish a shared language and understanding around teamwork. Alternatively, this could be provided to participants as a learning resource before or after participation in the scenario. We used the debriefing guide in Appendix E to guide discussion and provide formative feedback immediately after the simulation. Appendix F contains the didactic PowerPoint slides that were reviewed with participants before the simulation to solidify concepts related to the initial management of pediatric trauma. For participants with increased familiarity in the initial management of pediatric trauma, the didactic content in Appendix F could be presented during the debriefing session. We used the evaluation form in Appendix G to collect feedback from participants and assess the impact of the session on participant education. Appendix H includes a checklist of critical actions that should be performed by participants during the simulation.

### Assessment

Pediatric emergency medicine physicians facilitated and debriefed the scenarios as well as provided content expertise and feedback to participants mapped to the learning objectives. Small-group facilitators are all certified simulation instructors by our institution and provided formative feedback during the debrief on trauma, medical assessment, and the critical learning objectives, as is typical of formative learning simulation debriefs. Participants completed a survey (Appendix G) after the debriefing. Participants were asked to state their agreement with evaluative statements using a 5-point Likert scale (1 = *strongly disagree*, 2 = *disagree*, 3 = *neutral*, 4 = *agree*, 5 = *strongly agree*). They were asked about their experience participating in the session as well as their level of clinical confidence related to the learning objectives after participating in the simulation. They were also asked to answer two free-response questions related to their simulation experience: (1) “Describe one or more ways this session will change how you do your job” and (2) “How could we improve this simulation and workshop?”. Results from the two sessions were analyzed separately.

### Debriefing

The debriefing tools in Appendix E were used to facilitate debriefing sessions following the scenarios. These tools allowed facilitators to tailor the discussion based on observed knowledge gaps and performance of the participants. We began by allowing participants to provide general reflections of their experience, and then transitioned to a facilitated discussion of the components of the case. Observations made by participants and facilitators were used to transition into discussions on teamwork, communication, diagnostic skills, and management decisions. During the debriefing session, facilitators reviewed additional diagnostic tests in Appendix C if time permitted.

## Results

The first session contained 24 participants and the second session contained 25 participants. Participants included pediatric residents, medical students, and nurse practitioners. Four of the authors with training in pediatric emergency medicine facilitated both sessions. During the first session, groups took an average of 2.46 minutes to complete the primary survey (range: 0.83–4.00 min) and an average of 5.08 minutes to complete the secondary survey and provide a summary statement (range: 4.0–7.0 min). During the second session, groups took an average of 2.29 minutes to complete the primary survey (range: 1.00–3.92 min) and an average of 4.27 minutes to complete the secondary survey and provide a summary statement (range: 4.0–5.0 min).

Participants' level of agreement with statements related to their experience during the simulation sessions are summarized in Table 1. Table 2 summarizes participants' confidence with skills and knowledge related to pediatric trauma management after participation in the sessions.

**Table 1.**
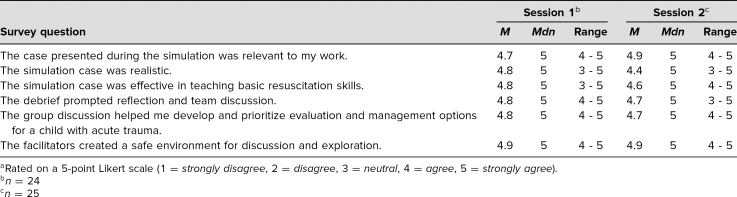
Participant Experience During Simulation^a^

**Table 2.**
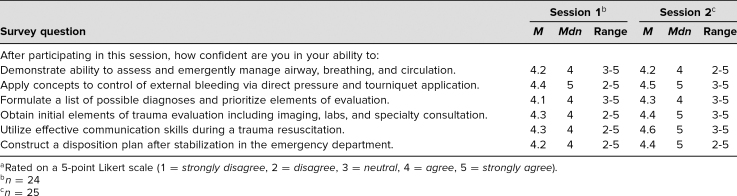
Participant Confidence After Simulation^a^

Below are themes and representative quotes obtained from participants' responses to the free-response survey question, “Describe one or more ways this session will change how you do your job.”

• Importance of structured/focused trauma survey
  ○ “Assess ABCDE whenever coming up on an unknown situation.”
  ○ “Perform initial ABC eval fast!”
  ○ “Remember to think about patients in a structured manner.”
  ○ “Help me focus my assessments.”
• Improved skills in trauma management
  ○ “Remember to examine whole patient without clothes to not miss major trauma.”
  ○ “Apply the tourniquet.”
  ○ “Check central pulse for circulation.”
• Increased learner comfort
  ○ “Feel more confident with primary survey.”
  ○ “More confident in a resuscitation situation.”
  ○ “I will be more confident approaching a child with an extremity bleed.”
  ○ “Given me confidence assessing trauma patients.”
  ○ “More prepared for trauma rotation.”
• Importance of communication/handoff skills
  ○ “Use my card to do ABCs in order more effectively and communicate better.”
  ○ “Good communication.”
  ○ “More efficient evaluation and handoff of trauma patients.”
• Review of existing clinical practice
  ○ “Always good to review steps for primary and secondary survey.”
  ○ “Great review of primary and secondary survey.”
  ○ “Primary survey practice is always helpful.”

Below are themes and representative quotes obtained from participants' responses to the free-response survey question: “How could we improve this simulation and workshop?”

• Increase time
  ○ “More time hands on!”
  ○ “I thought this was great. Just wish it was longer.”
  ○ “More simulations so everyone has a chance to practice airway, survey, and team lead.”
• Increase frequency of trauma education
  ○ “More often! Very useful practice – great hands on experience!”
  ○ “Do it more frequently!”
  ○ “More often please!”
  ○ “More of them throughout the year.”
• Increase realism
  ○ “Use a real person so they can role play true GCS, injury, mobility.”
• Increase discussion of medical management and skills
  ○ “More differential building.”
  ○ “Help improve my summary statement.”
  ○ “Summarize steps of trauma evaluation quickly right before and after simulation.”
• No changes
  ○ “Keep as is – this is great!”
  ○ “It was good!”
  ○ “This was great. I loved getting hands on practice!”
  ○ “Nothing, you all are great.”

## Discussion

This simulation case was designed for pediatric residents, who are generally novice to intermediate learners of the pediatric trauma assessment. The goal was to challenge participants to rapidly complete a structured pediatric trauma assessment and communicate their subsequent priorities in management while continuing to improve teamwork and communication skills. This simulation provides a safe learning environment for participants to rapidly evaluate simulated patients in the setting of a mass casualty event.

### Limitations

Limitations in the evaluation of this educational activity include that the scenario was tested on pediatric residents, medical students, and nurse practitioners. There may be aspects of this case which could be adapted to better serve other populations of health care workers, including physicians of other specialties, nursing students, physician assistants, and nurses who also care for this patient population. Mannequin limitations include not being able to simulate active bleeding/pallor, obvious deformity, or pain, which limited the realism of the scenario. However, this scenario aimed to identify appropriate treatment of trauma, which can be accomplished with moulage on a static mannequin. This curriculum was assessed primarily via Kirkpatrick's Level 1 (reaction), with not as much focus on Kirkpatrick's Level 2 (learning).^11^ Learners trended toward taking less time to complete the primary survey and summary statement in session two as compared to session one, which suggests that participant learning was positively affected. Performance could be measured more formally as learning (Kirkpatrick's Level 2) if this simulation was repeated with the same group of learners multiple times over a prolonged time period; however, this repetition was not feasible for our group of learners. Additionally, we used a convenience sample of providers at our institution, which may limit generalizability. Translation of knowledge acquired from this session to actual trauma mass casualty resuscitations was not measured, as this is an infrequently encountered event and would be difficult to complete logistically.

### Lessons Learned and Future Implications

There were several challenges regarding the simulation, one of which was time. Many participants commented that they would like additional time to practice the trauma assessment and/or review imaging, to have further discussion of differential diagnoses, and/or to repeat the trauma assessment on another patient with different team roles. A limitation was that our noon conference lasts only 45 minutes, but if the simulation were offered in a different setting, facilitators could devote additional time to these elements. During the first session, we noticed participants struggling to properly apply a cervical collar and log-roll patients, so discussion of these skills was added to the didactic portion for the second session. Another challenge we faced was distributing participants equally in number and level of experience to the four patients, which we addressed in the second session by having participants assigned to groups by facilitators. We did not run into space issues because we were able to utilize a large conference room that easily accommodated the four stations. This space was chosen because we anticipated 20–30 participants per session. Realism may be better preserved if the simulation is held in an ED, as that is the in situ space where care for mass casualty patients would usually occur. We suggest increasing the allowed time to 1 hour for the entire curriculum and recommend assigning participants to groups to achieve an equal number and skill mix of participants per group.

Our survey demonstrated that this curriculum improved participant comfort, confidence, and knowledge of pediatric trauma and was highly enjoyed. Survey comments suggest that this type of trauma education is highly desired by pediatric residents, which we suspect is not unique to our institution. Mass casualty events like the one presented in our scenario require high resource utilization but do not occur frequently, so simulation is a practical medium to practice these skills, particularly in institutions that are resident dependent. Ongoing pediatric trauma simulation may increase the comfort and confidence of novice learners during the low-frequency, high-risk event of pediatric mass casualty.

## Appendices

Simulation Case Template.docxSimulation Environmental Preparation.docxSimulation Images and Materials.pptxCommunication Tools.docxDebriefing Materials.docxDidactic PowerPoint Presentation.pptxEvaluation Form.docxCritical Actions Checklist.docx
All appendices are peer reviewed as integral parts of the Original Publication.
