# Supplementary material for: Pediatric Emergency Medicine Disaster Simulation Curriculum: The 5-Minute Trauma Assessment for Pediatric Residents (TRAP-5)
Source: MedEdPORTAL. 2020 Aug 21;16:10940. doi: 10.15766/mep_2374-8265.10940 (PMC7449578; doi:10.15766/mep_2374-8265.10940)
Supplement: Supplementary file 1 — Simulation Case Template.docxSimulation Environmental Preparation.docxSimulation Images and Materials.pptxCommunication Tools.docxDebriefing Materials.docxDidactic PowerPoint Presentation.pptxEvaluation Form.docxCritical Actions Checklist.docx [file mep_2374-8265.10940-s001.zip › A. Simulation Case Template.docx]

| Appendix A: MedEdPORTAL Simulation Case Template  SIMULATION CASE TITLE: Pediatric Disaster Simulation Series: The Five Minute Trauma Assessment for Pediatric Residents (TRAP-5)  AUTHORS: Tavis Dickerson-Young, MD, Ashley Keilman, MD, Hiromi Yoshida, MD, MBA, Maya Jones, MD, MPH, Nathan Cross, MD, MS, Anita Thomas, MD, MPH  LEARNER AUDIENCE: Beginning to intermediate learners of pediatric trauma such as pediatric residents | |
| --- | --- |
| PATIENT A: Brandon PATIENT B: Sofia  PATIENT AGE: 5 years PATIENT AGE: 5 years  CHIEF COMPLAINT: Right arm injury CHIEF COMPLAINT: Left leg injury  PHYSICAL SETTING: ED PHYSICAL SETTING: ED | |
|  | |
| Brief narrative description of case | In this scenario, pediatric residents are tasked with performing an initial trauma survey and triaging priorities in trauma management of pediatric patients in the setting of a disaster. Two scenarios are presented:  1) a long bone fracture with external hemorrhage and abdominal trauma  2) a long bone fracture with external hemorrhage and head trauma  This case may be repeated with a slightly different disaster scenario to assess learner retention of key concepts.  Anticipated interventions include assessment of the patient via primary survey, clear communication, anticipation of next two steps in trauma management which may include hemodynamic stabilization, labs/further imaging, and seeking expert consultation. The patient’s vital signs remain static throughout the case, and participants will have 5 minutes to complete their assessments. |
| Primary Learning Objectives | 1. Successfully perform primary survey within 2 minutes 2. Verbalize and prioritize next 2 steps in management within 5 minutes 3. Assign and maintain team roles 4. Demonstrate effective communication via clear closed-loop communication |
| Critical Actions | - Assessing patient via primary survey utilizing ABCDEs, noting other critical findings - Completion of assessment and concisely verbalize next steps within 5 minutes   - Obtain intravenous (IV) access and consider fluid/blood product resuscitation   - Head injury: Obtain imaging, cervical collar placement   - Abdominal injury: Obtain labs, imaging   - Long bone fracture: Obtain imaging   - Surgical consultation - Utilize clear communication and maintain team roles |
| Learner Preparation or Prework | - Didactic (Appendix F) - Pocket Primary Survey Card (Appendix C) - General knowledge of pediatric trauma   - Lavoie, M & Nance, M. (2016). Approach to the injured child. In K. Shaw & R. Bachur. (Eds.), *Fleisher & Ludwig’s textbook of pediatric emergency medicine* (pp. 9-19). Philadelphia, PA: Wolters Kluwer. |

| Initial Presentation | | | |
| --- | --- | --- | --- |
| Initial vital signs | Patient A: Heart rate (HR) 120, Oxygen saturation (SpO2) 98% on room air, Blood Pressure (BP) 100/65, Respiratory Rate (RR) 20, Temperature (T) 37 degrees Celsius  Patient B: Heart rate (HR) 120, Oxygen saturation (SpO2) 98% on room air, Blood Pressure (BP) 100/65, Respiratory Rate (RR) 20, Temperature (T) 37 degrees Celsius | | |
| Overall Setting and Appearance  *What do learners see when they first enter the room?* | The only people in the room initially are the facilitators and the participants with the goal of having all patients present at once. The instructor will facilitate the simulation by providing clinical updates or management suggestions if the team struggles. The facilitator can also play the role of the bedside nurse to obtain IV access, draw labs, or administer medications if requested by the participants.  Patient A: Brandon was brought from the bus accident for a right arm injury. He was found by medics lying on the ground next to the overturned school bus, moaning, eyes closed, not moving with bleeding from his right upper arm. He is carried from the scene on a stretcher by medics. Upon entering the room, the learner sees the patient lying on a backboard with bleeding from his right upper arm, his eyes open, crying, holding his right arm with his left hand. Blood soaked gauze obscures the open fracture with visible deformity. No parents or teachers are present.  Patient B: Sofia was brought from the bus accident for a left leg injury. She was found by medics lying on the ground next to the overturned school bus, moaning, eyes closed, not moving with bleeding from her left thigh. She is carried from the scene on a stretcher by medics. Upon entering the room, the learner sees the patient lying on a backboard with bleeding from her left thigh, her eyes open, crying, not moving her extremities. Blood soaked gauze obscures the open fracture with visible deformity. No parents or teachers are present. | | |
| Confederates and their roles in the room at case start  *Who is present at the beginning and what is their role? Who may play them?* | Each case should have at least three learners and one instructor.  Team Leader: Learner  Airway MD: Learner  Survey MD: Learner  **If there are other learners available, team leader may assemble them to expose the patient by removing clothing or assist with rolling the patient to fully assess for trauma.*  Instructor: Facilitates simulation and provides history. If the team is struggling with management, may provide suggestions in management, potentially assuming the role of an embedded participant such as a bedside nurse.  If other instructors are available, one may play the adult teacher who presents with the children and another may act as an embedded participant bedside nurse or embedded participant physician to assist in decision making if the team requires help. | | |
| HPI  *Please specify what info here and below must be asked vs. what is volunteered by the patient or other participants* | Patient A:  Brandon is a 5-year-old male on a field trip with his Kindergarten class when the bus overturned and he sustained a right arm injury. He will require a full trauma assessment.  *Start prebriefing just outside the room with the following instructions:*  “For this simulation, you will be the team assessing a patient from a multiple casualty accident. Your goal is to quickly assess the patient and determine the next steps in management. Specifically, you should aim to complete the primary survey within 2 minutes and in 5 minutes your team leader will provide a summary statement to the ED attending including your proposed next steps in management. Do you have any questions before we begin?”  *Once in the room provide the following information:*  “Your patient Brandon is a 20kg, 5-year-old male. He presented to the ED after being in a bus accident and sustained a right upper arm injury. He was noted by medics to be moaning, with eyes closed and not moving. Medics applied pressure with gauze to his bleeding right arm and carried him on the backboard.”  If asked about events leading up to the presentation (SAMPLE):  Signs/Symptoms: Bleeding from right upper arm, was in bus accident 15 minutes prior to arrival  Allergies: Unknown  Medications: Unknown  Past Medical History: Unknown  Last Meal: Unknown  Events Preceding: Unknown  Patient B:  Sofia is a 5-year-old female on a field trip with her Kindergarten class when the bus overturned and she sustained a left leg injury. She will require a full trauma assessment.  *Start prebriefing just outside the room with the following instructions:*  “For this simulation, you will be the team assessing a patient from a multiple casualty accident. Your goal is to quickly assess the patient and determine the next steps in management. Specifically, you should aim to complete the primary survey within 2 minutes and in 5 minutes your team leader will provide a summary statement to the ED attending including your proposed next steps in management. Do you have any questions before we begin?”  *Once in the room provide the following information:*  “Your patient Sofia is a 20kg, 5-year-old female. She presented to the ED after being in a bus accident and sustained a left leg injury. She was noted by medics to be moaning, with eyes closed and not moving. Medics applied pressure with gauze to her bleeding left leg and carried her on the backboard.”  If asked about events leading up to the presentation (SAMPLE):  Signs/Symptoms: Bleeding from left thigh, was in bus accident 15 minutes prior to arrival  Allergies: Unknown  Medications: Unknown  Past Medical History: Unknown  Last Meal: Unknown  Events Preceding: Unknown | | |
| Past Medical/Surgical History  Teacher believes that all the children are previously healthy | Medications  Unknown | Allergies  Unknown | Family History  Unknown |
| Patient A: Physical Examination | | | |
| General | Awake, holding his right arm, crying in pain | | |
| HEENT | Patent airway, 4 cm large boggy occipital hematoma without scalp bleeding, tympanic membranes are clear without hemotympanum, no battle sign, no nasal bleeding, dentition appears intact | | |
| Neck | Moving his head back and forth, cries with exam so unable to discern if there is cervical spine tenderness | | |
| Lungs | Clear breath sounds bilaterally. No stridor, crackles, or coarse breath sounds. No chest wall tenderness | | |
| Cardiovascular | Slightly tachycardic as per vitals above, regular rhythm, right arm radial pulse is 1+, other distal and central pulses are 2+, capillary refill 2-3 seconds, warm skin | | |
| Abdomen | Soft, non-tender, non-distended | | |
| Musculoskeletal | Right upper arm with approximately 3 cm laceration with spurting bleed, deformity indicative of underlying fracture | | |
| Neurological | Pupils are 3mm and reactive bilaterally, extraocular movements are intact, the patient has a Glasgow Coma Scale of 14 (eyes 4 opens spontaneously, verbal 4 crying, motor 6 follows commands) | | |
| Skin | Bruising on occiput as above and laceration on right arm | | |
| Genitourinary | Normal genitourinary exam | | |
| Psychiatric | Patient is alert, somewhat cooperative on exam, although crying and stating his arm hurts and that he is scared | | |
| Patient B: Physical Examination | | | |
| General | Awake, grabbing her left leg, crying in pain | | |
| HEENT | Patent airway, head is atraumatic with clear tympanic membranes and no hemotympanum, no battle sign, no nasal bleeding, dentition appears intact | | |
| Neck | Supple | | |
| Lungs | Clear breath sounds bilaterally. No stridor, crackles, or coarse breath sounds. No chest wall tenderness | | |
| Cardiovascular | Slightly tachycardic as per vitals above, regular rhythm, left leg dorsalis pedis pulse is 1+, other distal and central pulses are 2+, capillary refill 2-3 seconds, warm skin | | |
| Abdomen | Soft, 4 cm bruise on left upper quadrant (underneath clothes), tender to palpation with guarding | | |
| Musculoskeletal | Left thigh with approximately 3 cm laceration with spurting bleed, deformity indicative of underlying fracture | | |
| Neurological | Pupils are 3mm and reactive bilaterally, extraocular movements are intact, the patient has a Glasgow Coma Scale of 14 (eyes 4 opens spontaneously, verbal 4 crying, motor 6 follows commands) | | |
| Skin | Bruising on abdomen as above and laceration on left leg | | |
| Genitourinary | Normal genitourinary exam | | |
| Psychiatric | Patient is alert, somewhat cooperative on exam, although crying and stating her leg hurts and that she is scared | | |

| Instructor Notes - Changes and Case Branch Points  *This section should be a list with detailed description of each step that may happen during the case. If medications are given, what is the response? Do changes occur at certain time points? Should the nurse or other participant prompt the learners at given points? Should new actors or participants enter, and when? Are there specific things the patient will say or do at given times? There are a few examples given, but it is expected that most cases will have many more changes and potential branch points.* | | |
| --- | --- | --- |
| Intervention / Time point | Change in Case | Additional Information |
| *Prebriefing (outside the room, see HPI)* |  |  |
| *Facilitator and participants enter the room. Team should assign roles, designate team leader, and utilize clear communication amongst team members* | HR 120, SpO2 98% on room air, BP 100/65, RR 20, Temp 37 degrees Celsius | *Facilitator: “*For this simulation, you will be the pediatric team assessing a patient from a mass casualty incident. Your goal is to quickly assess the patient and determine the next steps in management. Do you have any questions before we begin?”  *If team does not assign roles within 2 minutes have facilitator assume role of bedside nurse and suggest “it may be helpful if we assign roles to organize our team and efficiently assess the patient, I’ll be the bedside nurse.* |
| *Prompt and systematic primary and secondary survey* | *Primary Survey:*  *A: Airway intact*  *B: Breathing intact, lungs clear to auscultation bilaterally*  *C: Note bleeding in extremity and implement hemorrhage control with direct pressure, packing, and/or tourniquet*  *D: Complete assessment with Glasgow Coma Scale call-out.*  *E: Expose the patient, rolling the patient for full visualization, and note head trauma (patient A) and abdominal trauma (patient B) and verbalize next steps*  *Secondary survey per physical exam.* | *If survey MD does not initiate primary survey within 1 minute or secondary survey within 3 minutes, facilitator as bedside nurse state to team leader “completing a prompt primary/secondary survey will help us to identify the patient’s injuries, it may be helpful if we had a team member perform the survey exam.”*  *If team focuses on bleeding rather than systematically going through ABCDEs, the facilitator or an embedded participant as bedside nurse can prompt the team with “What about the primary survey?” or “What about ABCDEs?”*  *If survey MD does not call out their exam loudly, facilitator as bedside nurse to say “it is helpful for you to call out your exam findings loudly and clearly as you go so that the rest of the team can hear and respond.”*  *If team does not apply direct pressure to the bleeding extremity or apply a tourniquet, the facilitator as bedside nurse may suggest this or offer to place a tourniquet.*  *If team does not expose patient, facilitator as bedside nurse “it may be helpful if we remove his/her clothes and log-roll to assess for additional injuries.”*  *If team notes concern for cervical spine injury and does not apply cervical spine immobilization, facilitator as bedside nurse may suggest this or offer to place cervical collar.* |
| *Identify and prioritize diagnostic tests* | *If asked for:*  *Venous Blood Gas: pH 7.38, pCO2 35, pO2 80, Bicarb 24, Base deficit 0*  *Glucose: 90*  *Point of care Hematocrit: 40*  **Trauma panel varies at different institutions, but may include a complete blood count (CBC), type and screen, type and cross, PT, PTT, INR, liver function tests, lipase, urinalysis, BUN, creatinine, electrolytes. (If asked for these values, instructor to state that they are pending)*  **If asked for imaging, instructor to state that portable x-ray is en route or that the team may prep and stabilize the patient to travel to computed tomography (CT)*  **If asked for specialists or supervising physician to come to bedside, instructor to state that these personnel are assisting with other patients from the mass casualty and will be there as soon as possible* | *Patient A: CT Head, x-rays of cervical spine and right humerus, trauma labs*  *Patient B: CT Abdomen/Pelvis, x-rays of left femur, trauma labs*  *Teams may consider additional imaging or labs as indicated.* |
| *Utilize clear and safe communication tools such as closed loop communication and summary statements*   \|  \| \| --- \| \|  \| |  | *Participant to use closed-loop communication.*  *Example: Team leader - “Survey MD, could you apply the tourniquet to the right arm/left leg.”*  *Survey MD - Tourniquet applied.*  *Team leader to provide summary statement to attending physician. Example Patient A: “This is a hemodynamically stable 5yo male who has an intact airway, a suspected skull fracture/intracranial bleed, hemorrhage from an open right humerus fracture controlled with a tourniquet who needs a head CT, right arm x-rays, trauma labs, neurosurgery and orthopedics consults.”*  *Example Patient B: “This is a hemodynamically stable 5yo female who has an intact airway, suspected abdominal trauma, hemorrhage from an open left femur fracture controlled with a tourniquet who needs an abdominal CT, left leg x-rays, trauma labs, general surgery and orthopedics consults.”* |

Ideal Scenario Flow

Provide a detailed narrative description of the way this case should flow if participants perform in the ideal fashion.

The learners are informed of the scenario outside of the room. As they enter the room, they are informed of each patient’s specific condition, focused on the long bone injury. Participants expediently assess the patient’s airway, breathing, circulation, disability, and exposure (ABCDE), noting that at C, the patient has active bleeding (patient A from the right upper extremity or patient B from the left lower extremity) and initiate hemorrhage control measures including applying direct pressure, packing, and/or tourniquet. While addressing this circulation issue, trainees may consider splinting, imaging of the extremity and obtaining expert consultation such as orthopedics. In exposure for patient A, participants should note the occipital hematoma and consider head trauma, thus stabilizing the cervical spine and requesting computed tomography of the head as well as neurosurgical consultation. In exposure for patient B, participants should note abdominal bruising and consider abdominal CT (+/- eFAST ultrasound) and general surgery consultation. Both patients require intravenous access, lab work, and participants may consider fluid or blood product resuscitation. The patient’s vitals remain static during this scenario. Participants may request repeat vital signs. The scenario concludes once the participant has completed their assessment, summarized the patient, and verbalized their next steps in management, or when 5 minutes has elapsed.

Anticipated Management Mistakes

Provide a list of management errors or difficulties that are commonly encountered when using this simulation case.

1. Failure to systematically assess the patient with primary survey followed by secondary survey: After being informed of the scenario, some learners may focus on the bleeding extremity as opposed to going through ABCDE in a systematic fashion. We found that reviewing the didactic before the simulation is most helpful to learners as well as reviewing and handing out the primary survey pocket card (Appendix C). Having a confederate prompt participants with suggestions such as “What about the airway/breathing/circulation/disability/exposure?” may also be helpful.
2. Failure of effective communication: While many participants were able to effectively summarize the patient, not everyone used commonly accepted communication tools. We found it helpful to review communication tools (Appendix D) before the simulation or during the debrief.
3. Failure to address other injuries: Most participants were able to recognize that the patients had a secondary injury of abdominal trauma or head injury. Most participants also recognized the need for cervical spine immobilization with suspected head injury. It may be helpful to review the importance of including exposure and spinal immobilization in the primary survey during the didactic or debrief. This was incorporated in our didactic and debrief. Recognition of additional injuries will help participants in formulation of next steps and disposition.
