# Supplementary material for: Pediatric Emergency Medicine Disaster Simulation Curriculum: The 5-Minute Trauma Assessment for Pediatric Residents (TRAP-5)
Source: MedEdPORTAL. 2020 Aug 21;16:10940. doi: 10.15766/mep_2374-8265.10940 (PMC7449578; doi:10.15766/mep_2374-8265.10940)
Supplement: Supplementary file 1 — Simulation Case Template.docxSimulation Environmental Preparation.docxSimulation Images and Materials.pptxCommunication Tools.docxDebriefing Materials.docxDidactic PowerPoint Presentation.pptxEvaluation Form.docxCritical Actions Checklist.docx [file mep_2374-8265.10940-s001.zip › B. Simulation Environmental Preparation.docx]

**Appendix B: Pediatric Disaster Simulation Environmental Preparation**

Before each simulation, ensure the anticipated resuscitation equipment is available for the team’s use.

**Resources**

PALS reference cards

Patient Weight Estimator (Broselow Tape)

Documentation forms

Primary Survey Pocket Card (Appendix C)

**Universal Precautions**

Staff gowns

Gloves

Mask and face shields

**Medications (consider having all or only a limited number of medications available)**

Acetaminophen

Amiodarone

Calcium Chloride

Calcium Gluconate

Dextrose

Epinephrine 1:10,000

Epinephrine 1:1,000

Etomidate

Fentanyl

Fosphenytoin

Hypertonic Saline (3%)

Ketamine

Levetiracetam

Lidocaine

Lorazepam

Midazolam

Normal Saline/Lactated Ringers

Ibuprofen

Rocuronium

Sodium Bicarbonate

Succinylcholine

**Equipment**

Mannequin in clothing, on bed with red liquid on injured extremity

Monitor – NIBP, HR, RR, oxygen saturation, temperature and ETCO2 monitor (if available)

Blood pressure cuff, heart rate monitor leads, oxygen saturation probe, defibrillator cables,

and ETCO2 cannula (if available)

Oxygen hook-up on wall or cylinder

Bag-mask system, multiple size masks

O_2_ – nasal cannula, mask - simple and/or non-rebreather

Suction

Thermometer, temperature probe

Nasal, oral airways, multiple sizes

Shoulder roll

Endotracheal tubes- 3.0, 3.5, 4.0, 4.5, 5.0, cuffed or uncuffed, stylets

Laryngoscope, Miller and Mac blades, multiple sizes

End-tidal CO2 colorimeter

Nasogastric tube(s)

Stethoscopes

IV/Angiocath, various sizes

IO needles

Gauze, Tape

IV tubing/blood product tubing and filters, stopcock

IV pumps, pressure bags/ blood product pumps

Syringes, multiple sizes

Bedside blood sample processors: glucose, electrolytes, gases

Specimen tubes

Crash cart & backboard

Defibrillator / AED

Tourniquet and gauze for packing bleeding wounds

Packed red blood cells

Cervical spinal collar

Penlight

Stopwatch (for facilitator use)
