# Supplementary material for: Pediatric Emergency Medicine Disaster Simulation Curriculum: The 5-Minute Trauma Assessment for Pediatric Residents (TRAP-5)
Source: MedEdPORTAL. 2020 Aug 21;16:10940. doi: 10.15766/mep_2374-8265.10940 (PMC7449578; doi:10.15766/mep_2374-8265.10940)
Supplement: Supplementary file 1 — Simulation Case Template.docxSimulation Environmental Preparation.docxSimulation Images and Materials.pptxCommunication Tools.docxDebriefing Materials.docxDidactic PowerPoint Presentation.pptxEvaluation Form.docxCritical Actions Checklist.docx [file mep_2374-8265.10940-s001.zip › D. Communication Tools.docx]

**Appendix D: Pediatric Disaster Simulation Communication Tools**

| Term | Definition |
| --- | --- |
| Call-Out | A tactic used to communicate critical information during an emergent event. Helps the team prepare for vital next steps in patient care. *(Example: “Airway status?” – “Airway clear”; “Breath sounds?” – “Breath sounds decreased on right”)* |
| Check-Back or Closed Loop Communication | Verification of communicated information. A message is initiated, then restated by the intended recipient, and that restatement is acknowledged/verified by the sender. *(Example: “Give a 20 mL/kg normal saline bolus IV push” – “20 mL/kg normal saline IV push” – “That’s correct”)* |
| CUS | Utilizing the key phrases “I am Concerned, I am Uncomfortable, this is a Safety issue” in order to clearly communicate the gravity of the issue raised. |
| Debrief | An informational/educational session designed to improve participant performance in the scenario. |
| Huddle | Ad hoc planning to re-establish situational awareness; designed to reinforce plans already in place and assess the need to adjust the plan. |
| Pre-Brief | A brief discussion prior to the start of the scenario to assign roles, establish expectations and expected outcomes. |
| QVV | When unsure of next steps, qualify the source, validate the source, and verify understanding. |
| SBAR | An outline of succinct communication. S = Situation (What is the patient’s situation?) B = Background (What is the context?) A = Assessment (What is the issue?) R = Recommendation (What would I recommend to correct it?) |
| Shared Mental Model | An organizing knowledge structure of relevant facts and relationships about a task or situation that are commonly held by team members |
| Situational Awareness | The ability to identify, process, and comprehend the critical elements of information about what is happening to the team with regards to the mission. It’s knowing “What is going on around you” and “What is likely to happen next.” |

Adapted from:

Bartman T, McCLead RE. *Core Principles of Quality Improvement and Patient Safety*. Pediatrics in Review Oct 2016, 37 (10) 407-417; DOI: 10.1542/pir.2015-0091

U.S. Department of Health and Human Services (HHS) Agency for Healthcare Research and Quality (AHQR) and Department of Defense (DoD) Team Strategies & Tools to Enhance Performance & Patient Safety (TeamSTEPPS): *TeamSTEPPS 2.0. Content last reviewed August 2018. Agency for Healthcare Research and Quality, Rockville, MD.* [*http://www.ahrq.gov/teamstepps/instructor/index.html*](http://www.ahrq.gov/teamstepps/instructor/index.html)

Weick KE, Sutcliffe KM. *Managing the Unexpected: Resilient Performance in an Age of Uncertainty.* San Francisco, CA: Jossey-Bass; 2007
