# Supplementary material for: Pediatric Emergency Medicine Disaster Simulation Curriculum: The 5-Minute Trauma Assessment for Pediatric Residents (TRAP-5)
Source: MedEdPORTAL. 2020 Aug 21;16:10940. doi: 10.15766/mep_2374-8265.10940 (PMC7449578; doi:10.15766/mep_2374-8265.10940)
Supplement: Supplementary file 1 — Simulation Case Template.docxSimulation Environmental Preparation.docxSimulation Images and Materials.pptxCommunication Tools.docxDebriefing Materials.docxDidactic PowerPoint Presentation.pptxEvaluation Form.docxCritical Actions Checklist.docx [file mep_2374-8265.10940-s001.zip › E. Debriefing Materials.docx]

**Appendix E: Pediatric Disaster Simulation Debriefing Guide**

**Debriefing Overview**

*Simulation creates the opportunity to debrief. We believe that the focus of each simulation should be the DEBRIEF. Simulation creates the opportunity to examine our medical management, technical skills, teamwork, and communication skills. It facilitates discussion about challenges in a safe environment in order to improve the quality of patient care.*

**Framework for debriefing:**

Each debrief should consist of 5 components:

- - - Introduction
    - Discussion of emotions
    - Discussion of medical management and technical skills
    - Discussion of teamwork and communication skills
    - Summary

*There is often overlap between medical management and teamwork issues. Debriefing may not follow a linear progression of all four of these components.*

General Debriefing Goals:

- Try to facilitate the **team’s** discussion (avoid dominating the conversation)
- Ask open ended questions (avoid yes/no questions)
- Discuss the team performance (not the individual)

1. **Introduction**

This “sets the stage” for debriefing and creates expectations. What you might say:

- This is an opportunity to reflect and learn, improve our medical care, teamwork, and communication.
- Everyone should be able to ask questions and share their thoughts.
- Once you leave this session, we encourage open discussion of the concepts, but ask you to not to discuss individual performance.
- Remind the group of the ground rules to treat everyone with respect and maintain confidentiality.

1. **Discussion of Emotions**

There are a couple of camps regarding discussing emotions. One perspective is that until emotions are dealt with, it’s difficult for adult learners to “move on” or switch gears to process thoughts, actions, and opportunities for improvement. Another perspective is that adult learners should process their emotions independently.

Our perspective is the first. If a group or team member is emotionally charged (sad, mad, or frustrated) regarding something that did or didn’t happen in a scenario, it’s usually difficult for the individual or the group to be actively engaged, receptive to feedback, and able to promote learning until the emotions are addressed. Validate the reactions and emotions of the learner. If learners don’t volunteer their reactions or emotions, you may suggest some common ones.

What you might say:

- These situations can be intimidating…

An example: a medication error occurs. One team member may think it is their fault. They may feel embarrassed, judged, etc. If they can verbalize this, other team members may offer different perspectives which enable the team to process the error together, potentially identifying contributing system issues. If the emotions aren’t addressed, people may feel embarrassed or responsible and not engage in a discussion, failing to identify system issues which led to the error.

What you might say:

- How did that feel?
- Can you tell me more?
- Why?

1. **Discussion of Medical Management and Technical Skills**

This portion of the discussion focuses on the medical aspects of the scenario. It may be more comfortable to begin with these “facts”.

What you might say:

- - - Let’s begin by discussing medical management.
    - What did you think was wrong with the patient?
    - Can someone briefly summarize what happened in this scenario?
    - How did you reach those conclusions?

1. **Discussion of Teamwork and Communication Skills**

This portion of the discussion focuses on how the team worked together. It can be emotionally charged and difficult to discuss without feeling personal. The challenge is to try to generalize specifics into themes.

What you might say:

- - - Let’s talk about how you functioned as a team.
    - What did your team do well?
    - What could your team do differently next time?
    - That is something I see often. Has anyone else experienced that? How have you seen that handled?

### 5) Summary

- This is your opportunity to ensure the key learning points are highlighted
- Try to identify approximately three take-home points
- You may ask the participants’ to identify take home points or call them out yourself.

*Medical management/technical skills examples:*

- - - - 1. This was a scenario of pediatric trauma in the setting of disaster.
        2. Potential injuries in the pediatric trauma patient are countless. Thus, evaluation and management of pediatric trauma should systematically begin with a primary survey and assessment of vital signs.
        3. Other key elements of pediatric trauma include rapidly obtaining IV access, obtaining and interpreting labs/imaging, anticipating need for additional interventions, and seeking expert consultation.

*Teamwork/communication examples:*

- - - - 1. Recognize need for a full resuscitation team in the management of pediatric trauma.
        2. Designate leadership and team member roles to ensure coordinated team functioning.
        3. Use *brief* or *huddle* to create a shared mental model for the working diagnosis and management plan.

**Debriefing Guide**

Below are examples of learning objective based statements & questions you may use to debrief the team.

| **Examples of debriefing for different learning objectives** | | |
| --- | --- | --- |
| **Perform a primary survey for patient with trauma** | | |
| Debriefer Script | Reference Material | Instructor Notes |
| I noticed you *(were complete/missed some opportunities)* in performing the primary survey. This was *(great/could have been even better)* because early identification and management of injury can lead to improved outcomes*.*   - How did your team decide on the evaluation priorities? - What helped/hindered you?   I see you *(were quick/took a while)* to include abdominal/intracranial/ extremity trauma on your differential diagnosis. This *(was great/could have been even better)* since delays in recognition can result in clinical deterioration.   - What were your thought processes around what was occurring? - What helped/hindered you in systematically assessing the patient? - (If evaluation did not begin with primary survey) How did you determine where to begin your assessment? | Components of an initial evaluation   - Primary survey (ABCDE) - Vital signs   Primary Survey Badge/Pocket Card (Appendix C) |  |
| **Identify and prioritize diagnostic tests** | | |
| Debriefer Script | Reference Material | Instructor Notes |
| I noticed you *(were quick/took a while)* to identify possible injuries and order trauma labs or CT/XR imaging. This was (*great/could lead to delays)* since delays in recognition can result in clinical deterioration.   - What were your thought processes around what was occurring? - What helped/hindered you in deciding what diagnostic tests to order? | Initial management of pediatric trauma patient   - Trauma labs - Trauma series (AP XR of the chest and pelvis) - FAST US (if available) - Additional imaging as appropriate based on injuries/mechanism - Subspecialty consultation |  |

| **Examples for debriefing different teamwork learning objectives** | | | |
| --- | --- | --- | --- |
| **Roles and responsibilities** | | | |
| Debriefer Script | Reference Material | | Instructor Notes |
| Let’s talk about how you functioned as a team.  From my perspective it looked like you (*did/did not)* have a clear team leader and defined team roles. I think this is (*great/concerning)* because clear team roles can help a team function smoothly, improve how quickly interventions take place, and reduce errors.   - How did you function as a team? - What did you think about your roles? | Team leader   - Establish self as leader, give clear direction, coordinate timely interventions - Stands at the foot of patient throughout resuscitation   Airway MD   - Manage airway - Stands at head of patient   Survey MD   - Primary and secondary survey, palpate pulses, reassessments   Nursing roles   - Medication preparation - Medication administration - Documentation | |  |
| **Brief and huddle** | | | |
| Debriefer Script | | Reference Material | Instructor Notes |
| I noticed that your team *(did/didn’t/took a while to)* (*brief* *prior to the initial patient assessment/huddle after the initial evaluation).* I thought this was (*great/could have helped you work better as a team*) in order to facilitate patient care.   - What *(helped/hindered)* your team from (*briefing/huddling*)? - How did that impact your team? - What could your team have done differently? - How can you make sure that *(does/doesn’t*) happen again? | | The goal of a brief/huddle is to create a shared mental model. A brief or huddle can assure all team members know what the working diagnosis is, and what the management priorities and next steps in care are.   - Everyone on the team is responsible for making this happen. Anyone can ask for a brief/huddle. Brief/huddle is usually led by the team leader. - If one team member doesn’t know what’s happening or what the management priorities are, they are probably not alone. |  |

| **Directed call out** | | | |
| --- | --- | --- | --- |
| Debriefer Script | Reference Material | | Instructor Notes |
| I noticed that you (*did/didn’t/intermittently*) used (*peoples names/roles/eye contact*) when (*calling out orders/asking for assistance*). I thought this was (*great/could have been more directed*) in order to facilitate communication.   - What did you notice about orders/questions that were asked? - How did this impact your team? | Directed call out is a tactical communication skill to assure that important orders/questions are specifically directed to one individual (rather than called out into the air).   - “Jonathan, what’s the SaO2?” - “Kim, give 500mL normal saline” - “Team leader, she stopped responding to pain” | |  |
| **Closed loop communication/check back** | | | |
| Debriefer Script | | Reference Material | Instructor Notes |
| I noticed that you used closed-loop communication *(consistently/a lot/rarely)*. Closed-loop communication can be critical for catching errors and assuring that *(information/an order/a request)* is heard.   - How were the communication loops in the team? - How did that impact your team? - Has anyone seen problems with this in a patient resuscitation? - Has anyone seen closed loop communication prevent an error? - How could you do it differently next time? | | Closed loop communication/check back is a strategy that requires verification of information. This enables the sender of the message to verify it has been heard and heard correctly. It enables the receiver to confirm what they heard is correct.   - Team leader “Call for CT” - Float nurse “Calling technician to confirm availability of the CT scanner” - Team leader “Thank you” |  |

**Pediatric Disaster Simulation Medical Management Debriefing Form**

This checklist identifies core medical management/technical skills. It’s hard to discuss more than 3 of these during one debriefing session. We recommend focusing on 2-3 of these issues.

**Primary survey □** Done Well **□** Needs Work

Specific comments:

*Discussion Points: What did you think of the initial evaluation of this patient? What could you do differently? What else should you consider on your differential?*

**Secondary survey □** Done Well **□** Needs Work

Specific comments:

*Discussion Points: What did you think of the additional evaluation of this patient? What could you do differently? What else should you consider on your differential?*

**Identify and prioritize diagnostic tests □** Done Well **□** Needs Work

Specific comments:

*Discussion Points: How long did it take you to recognize and prioritize the initial tests for this patient? What (went well/could have gone better)?*

**Pediatric Disaster Simulation Teamwork and Communication Debriefing Form**

This checklist identifies core teamwork and communication skills. It’s hard to discuss more than 3 of these during one debriefing session. We recommend focusing on 2-3 of these issues.

**Team roles identified and maintained □** Done Well **□** Needs Work

Specific comments:

*Discussion Points: What helped/hindered having clear leadership and roles?*

**Shared mental model □** Done Well **□** Needs Work

Specific comments:

*Discussion Points: How did team members share information or arrive at a working diagnosis/management plan (brief/huddle)?*

**Directed call out □** Done Well **□** Needs Work

Specific comments:

*Discussion Points: Were orders given “into the air” or directed at specific individuals? How did that impact you? How could they be delivered more effectively?*

**Closed loop communication and check back □** Done Well **□** Needs Work

Specific comments:

*Discussion Points: Describe use and effectiveness of closed loop communication.*
