# Supplementary material for: Pediatric Emergency Medicine Disaster Simulation Curriculum: The 5-Minute Trauma Assessment for Pediatric Residents (TRAP-5)
Source: MedEdPORTAL. 2020 Aug 21;16:10940. doi: 10.15766/mep_2374-8265.10940 (PMC7449578; doi:10.15766/mep_2374-8265.10940)
Supplement: Supplementary file 1 — Simulation Case Template.docxSimulation Environmental Preparation.docxSimulation Images and Materials.pptxCommunication Tools.docxDebriefing Materials.docxDidactic PowerPoint Presentation.pptxEvaluation Form.docxCritical Actions Checklist.docx [file mep_2374-8265.10940-s001.zip › F. Didactic PowerPoint Presentation.pptx]

## Slide 1
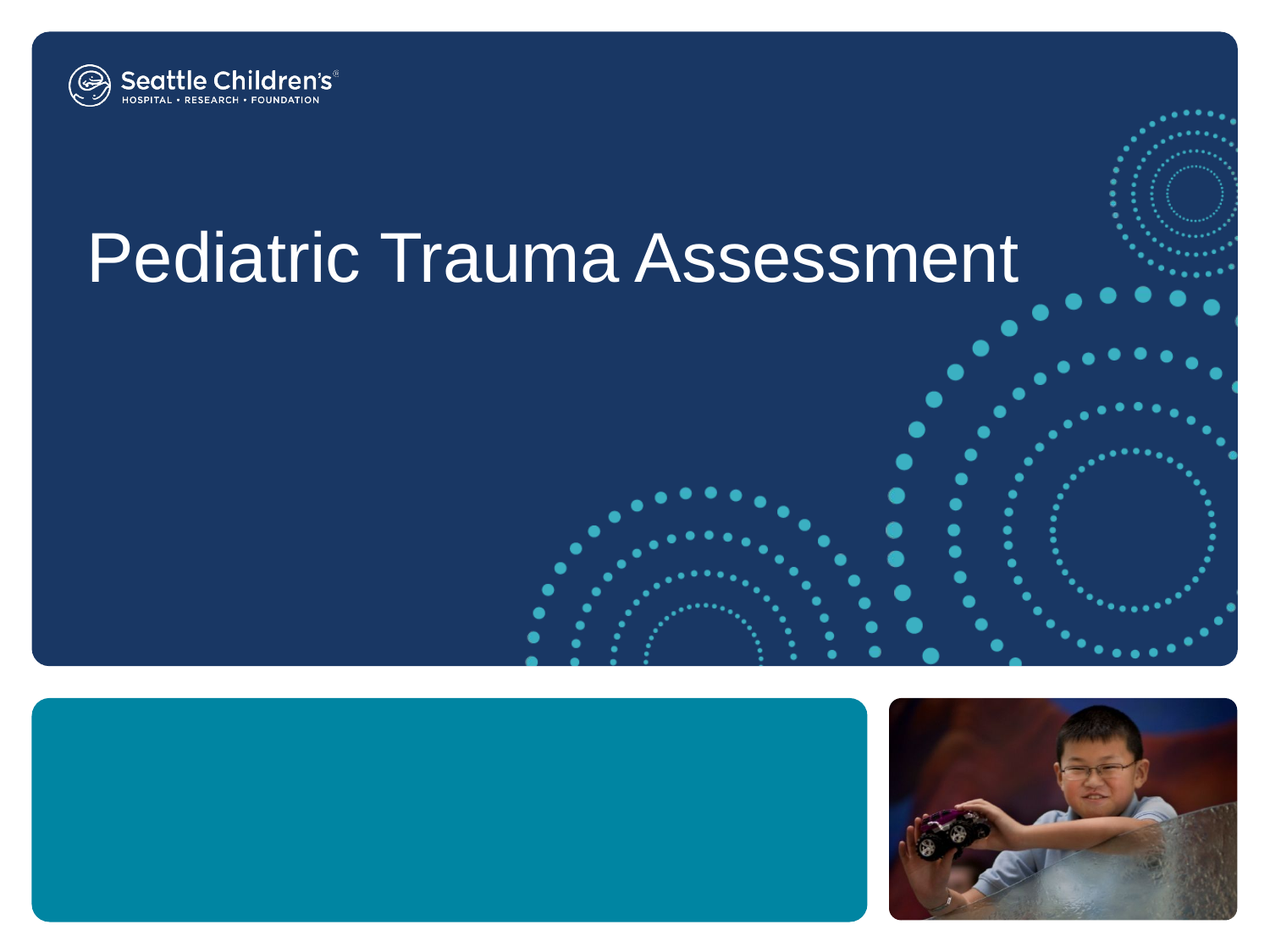

# Pediatric Trauma Assessment

## Slide 2
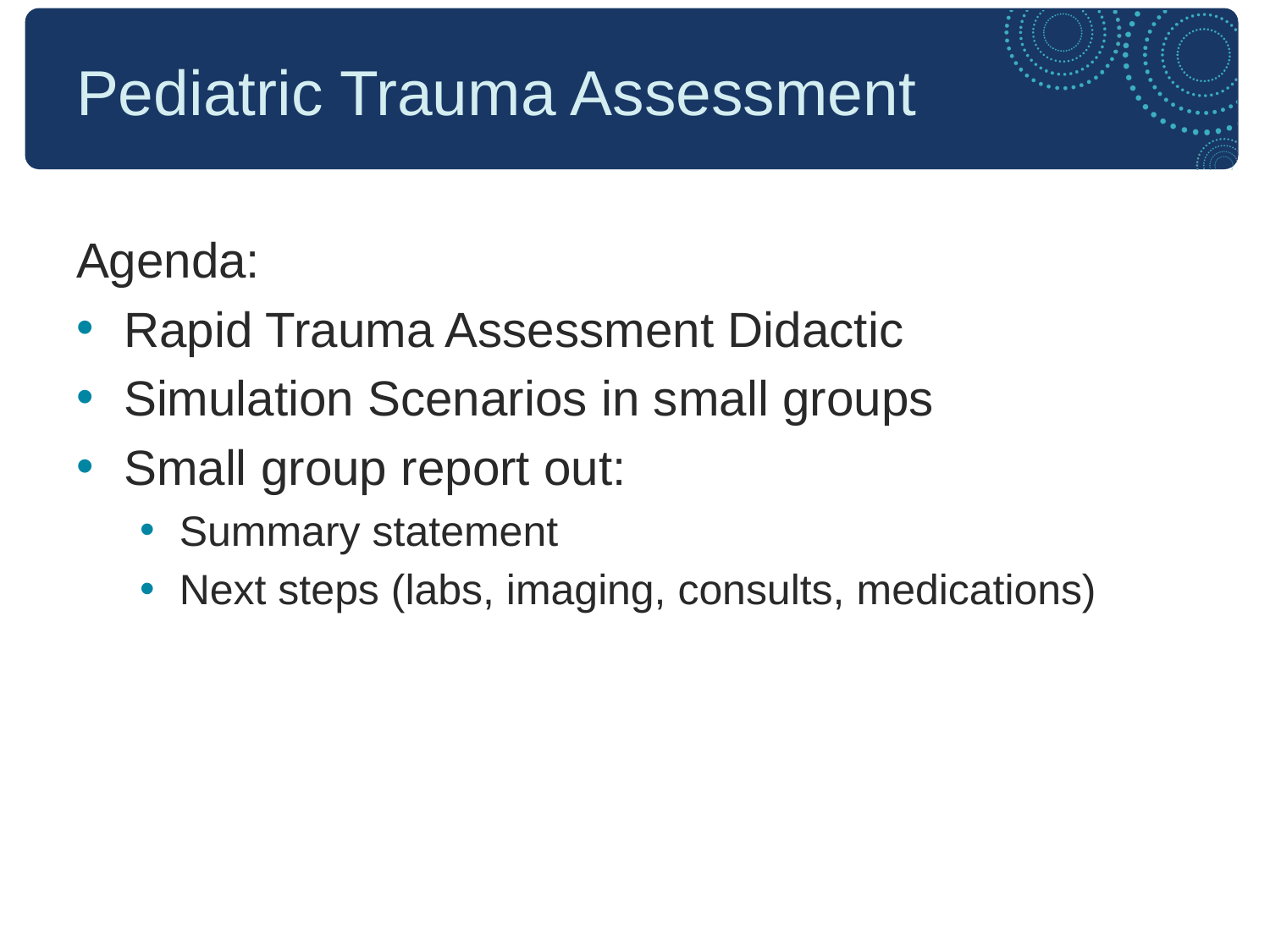

# Pediatric Trauma Assessment
Agenda:
Rapid Trauma Assessment Didactic
Simulation Scenarios in small groups
Small group report out:
Summary statement
Next steps (labs, imaging, consults, medications)

## Slide 3
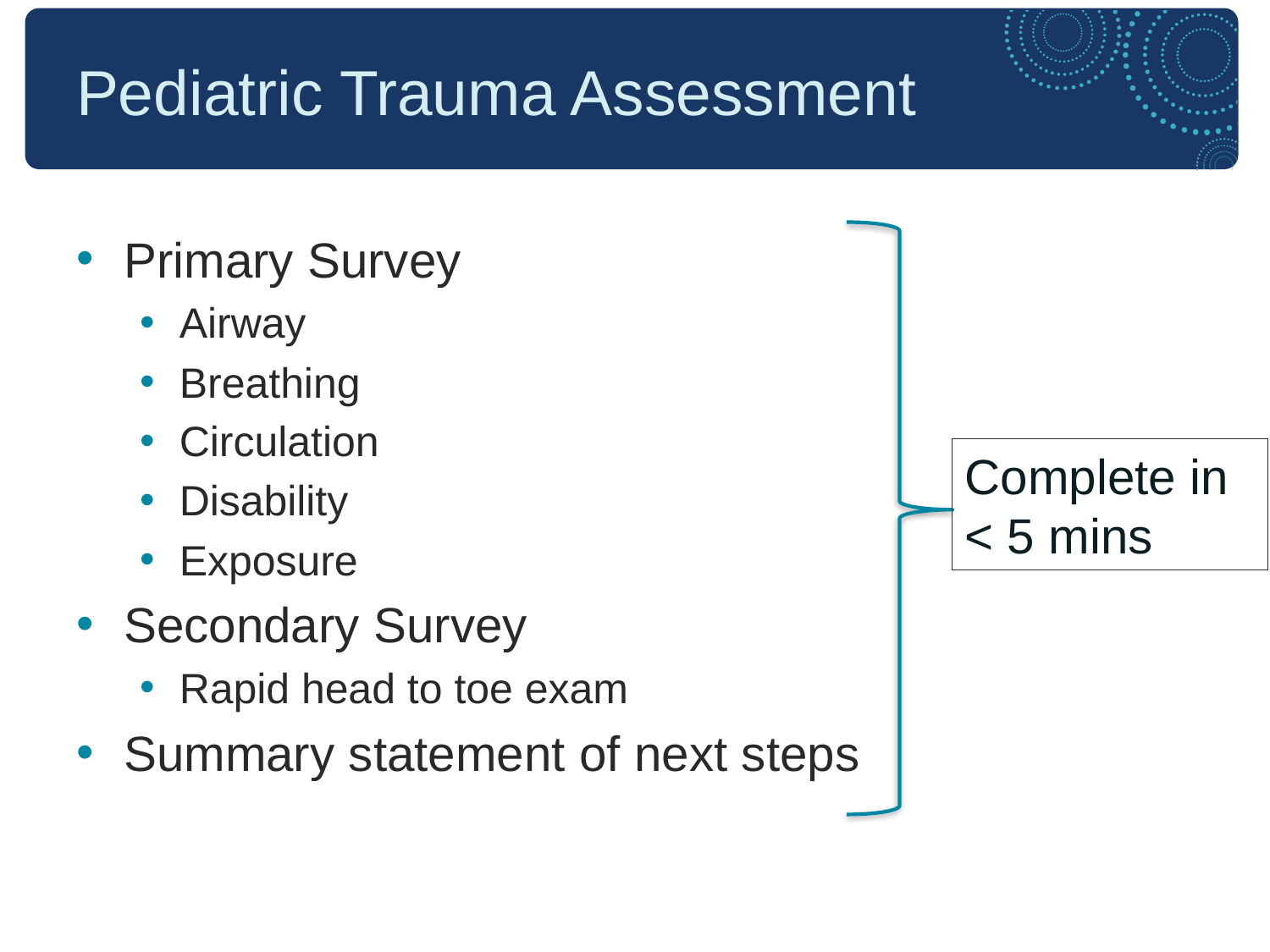

# Pediatric Trauma Assessment
Primary Survey
Airway
Breathing
Circulation
Disability
Exposure
Secondary Survey
Rapid head to toe exam
Summary statement of next steps
Complete in
< 5 mins

## Slide 4
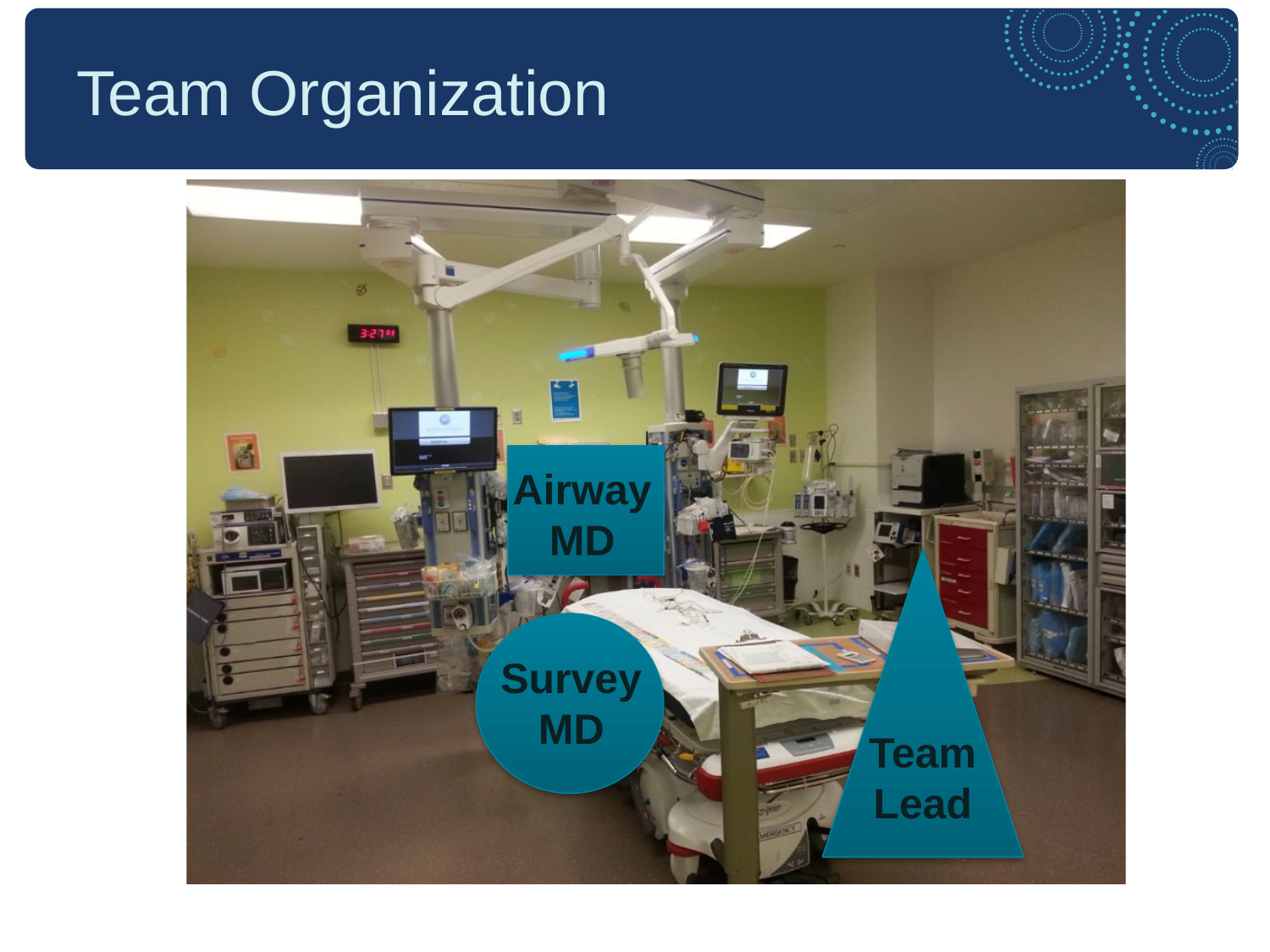

# Team Organization
Airway MD
Survey MD
Team Lead

## Slide 5
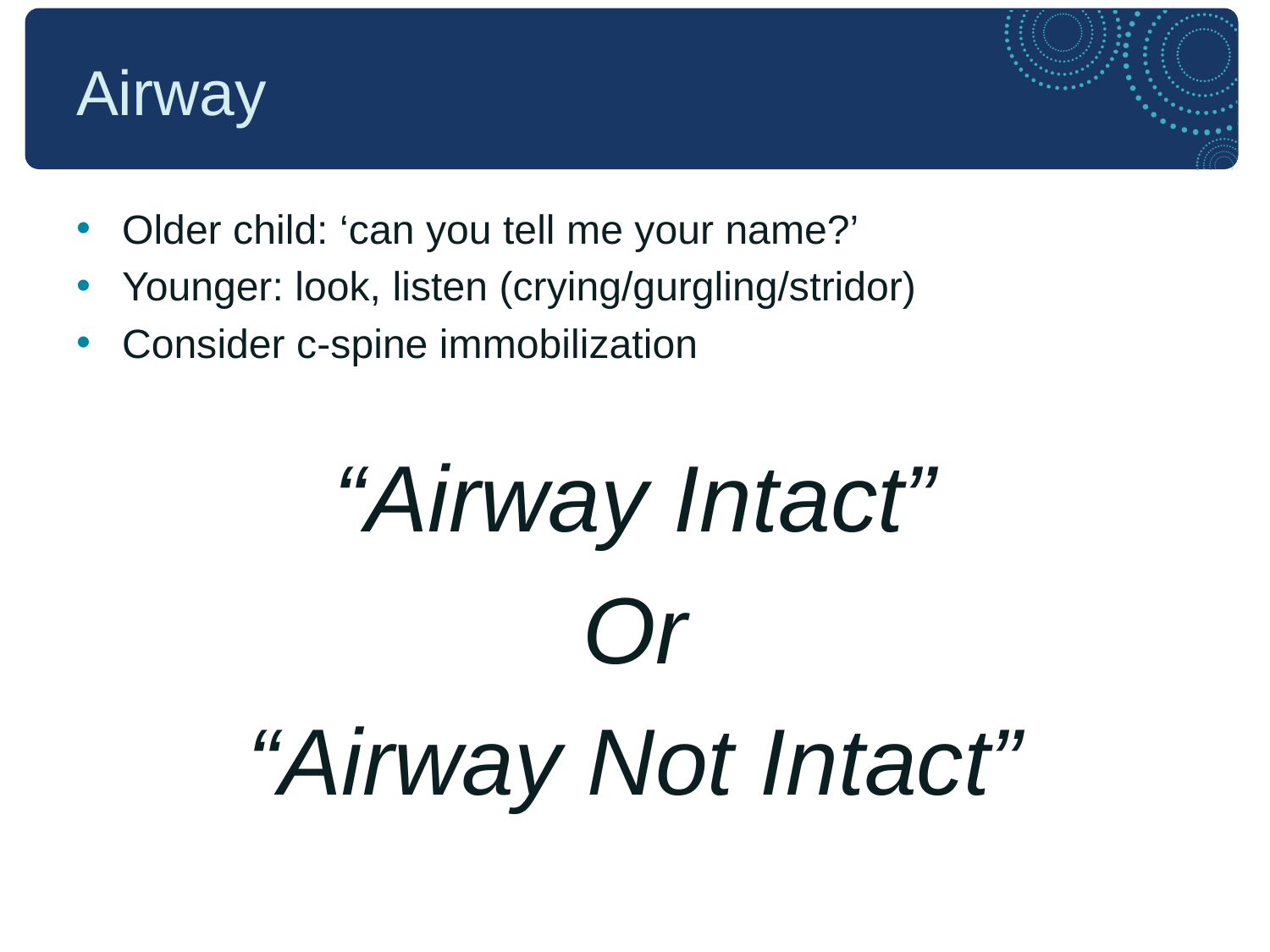

# Airway
Older child: ‘can you tell me your name?’
Younger: look, listen (crying/gurgling/stridor)
Consider c-spine immobilization
“Airway Intact”
Or
“Airway Not Intact”

## Slide 6
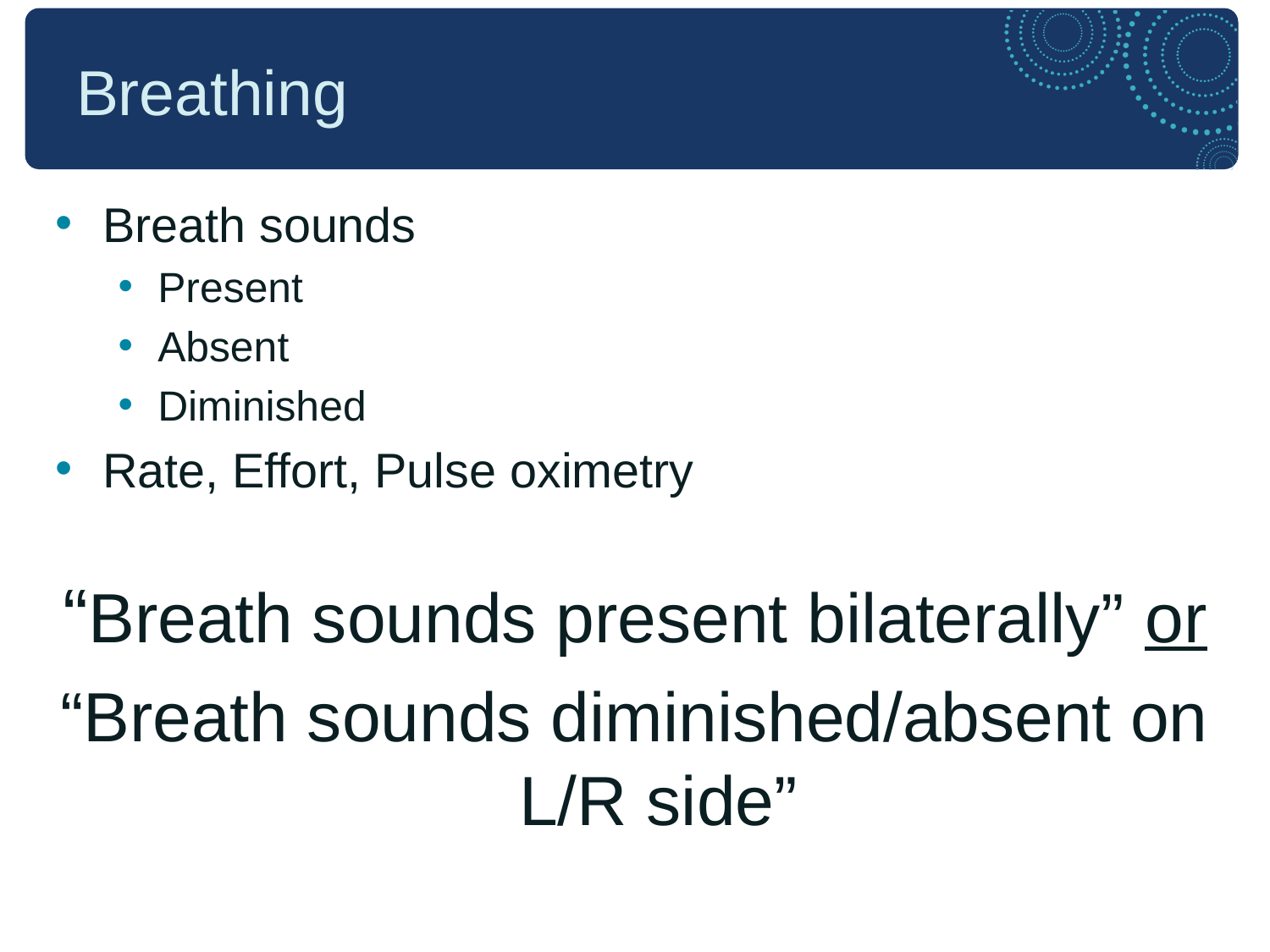

# Breathing
Breath sounds
Present
Absent
Diminished
Rate, Effort, Pulse oximetry
“Breath sounds present bilaterally” or
“Breath sounds diminished/absent on L/R side”

## Slide 7
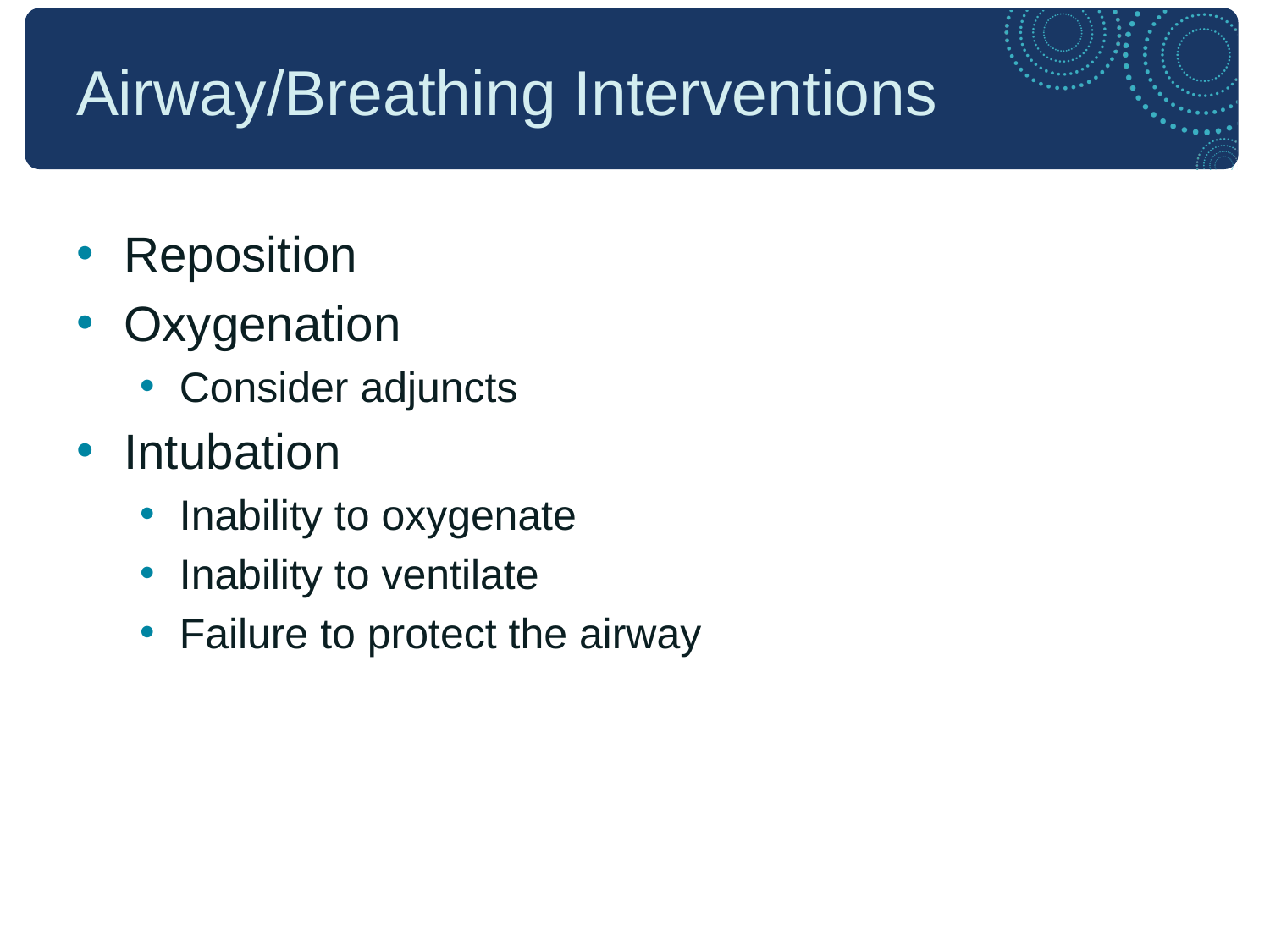

# Airway/Breathing Interventions
Reposition
Oxygenation
Consider adjuncts
Intubation
Inability to oxygenate
Inability to ventilate
Failure to protect the airway

## Slide 8
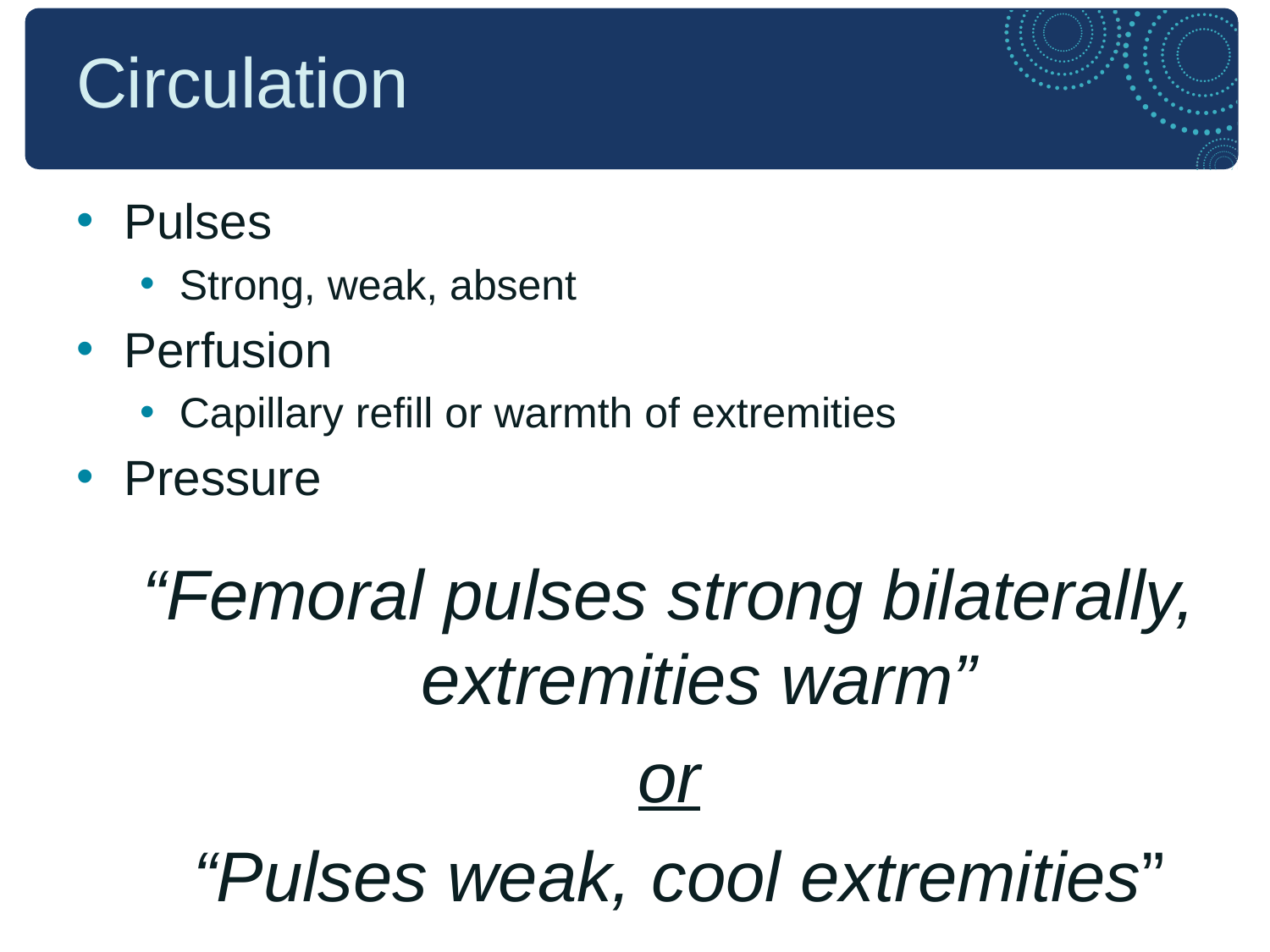

# Circulation
Pulses
Strong, weak, absent
Perfusion
Capillary refill or warmth of extremities
Pressure
“Femoral pulses strong bilaterally, extremities warm”
or
“Pulses weak, cool extremities”

## Slide 9
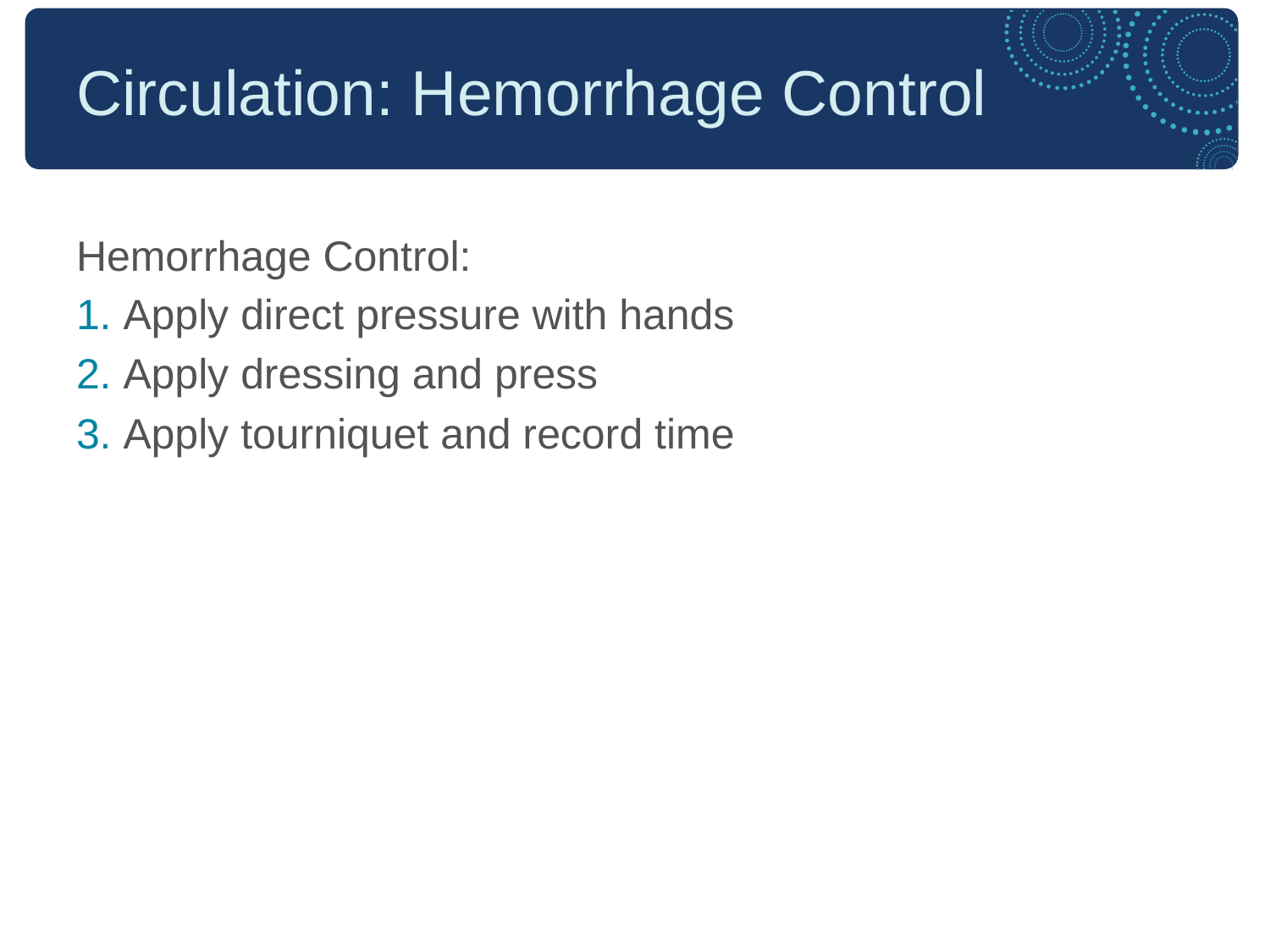

# Circulation: Hemorrhage Control
Hemorrhage Control:
 Apply direct pressure with hands
 Apply dressing and press
 Apply tourniquet and record time

## Slide 10
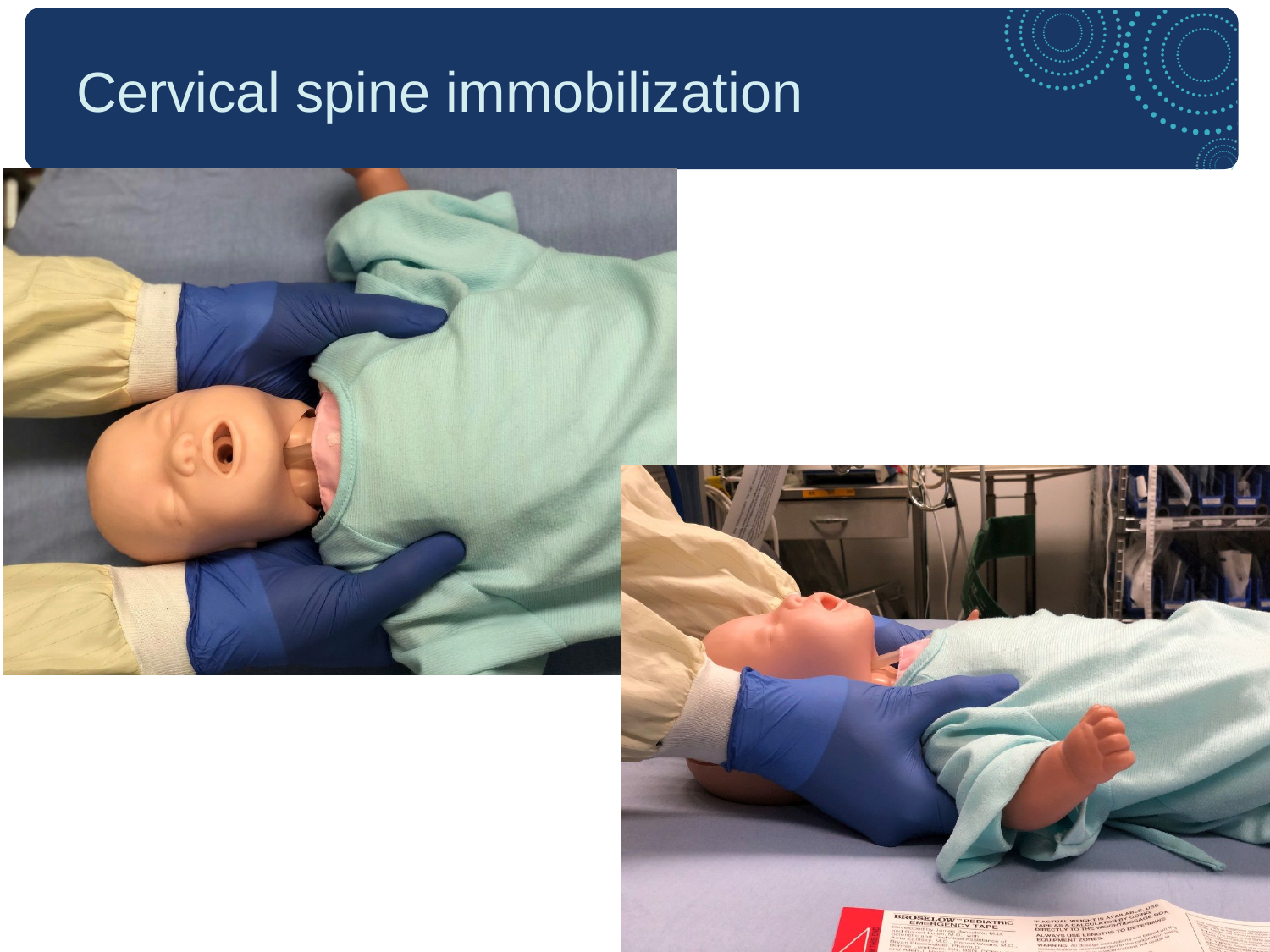

# Cervical spine immobilization

## Slide 11
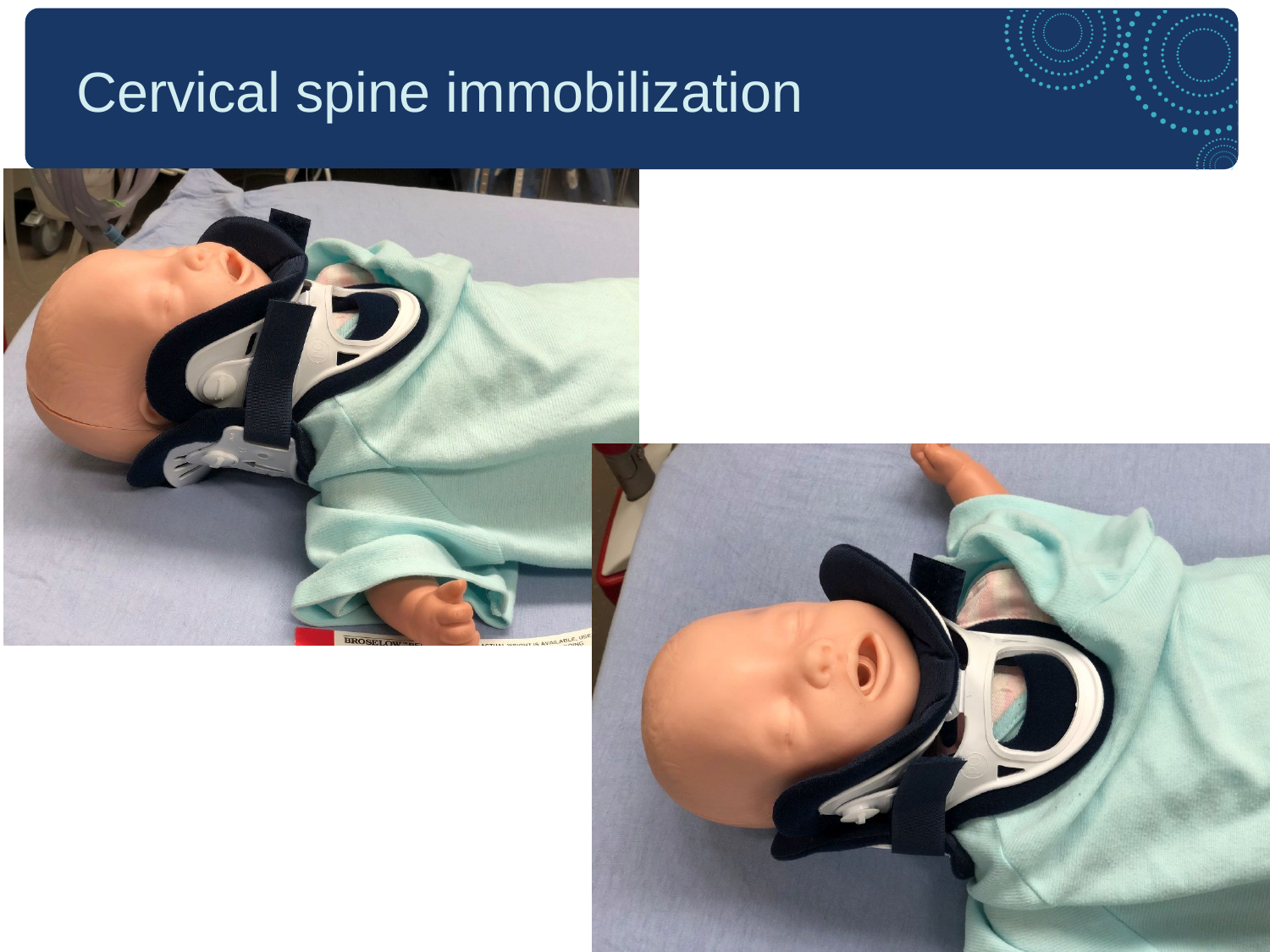

# Cervical spine immobilization

## Slide 12
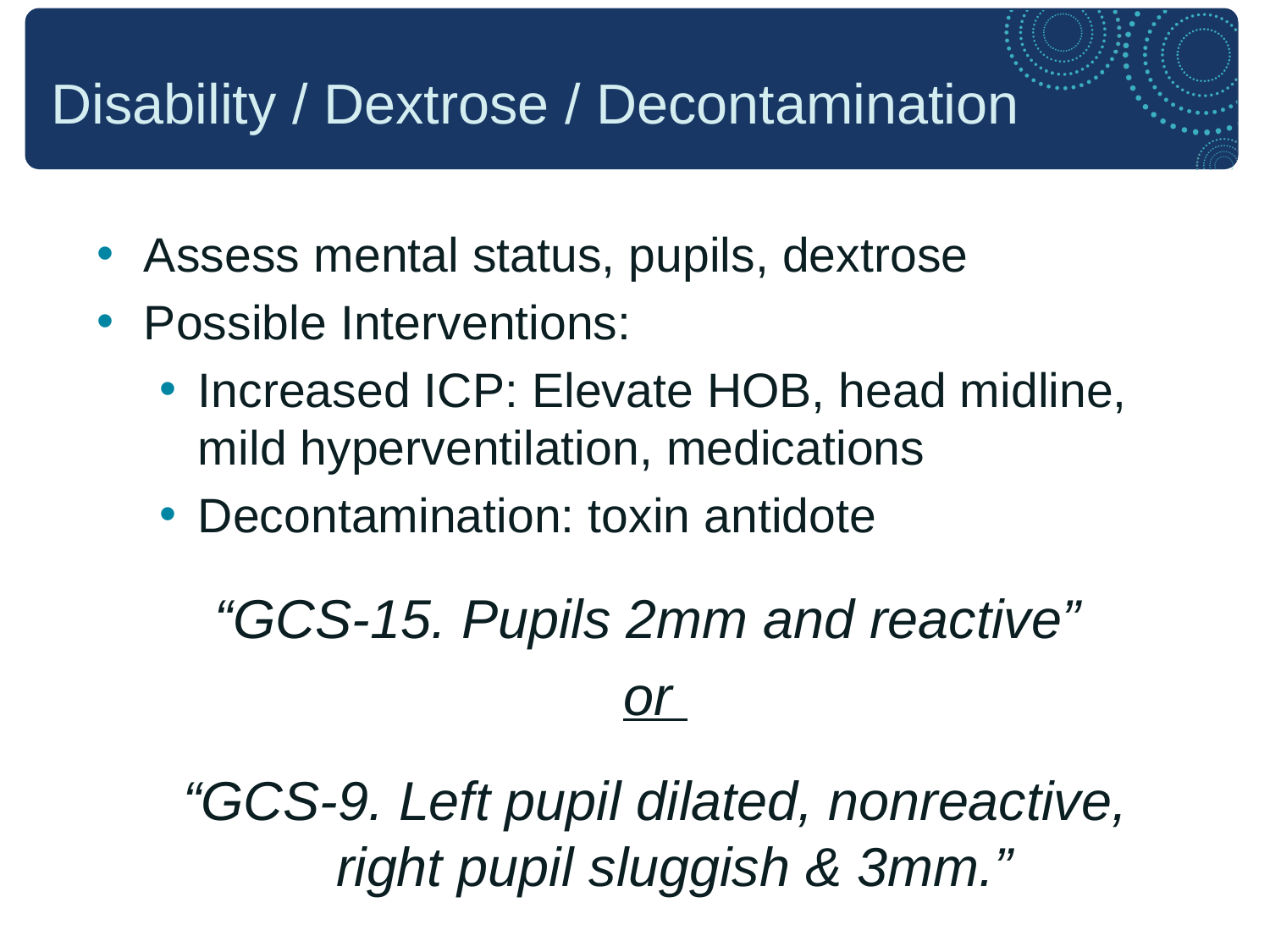

# Disability / Dextrose / Decontamination
Assess mental status, pupils, dextrose
Possible Interventions:
Increased ICP: Elevate HOB, head midline, mild hyperventilation, medications
Decontamination: toxin antidote
“GCS-15. Pupils 2mm and reactive”
or
“GCS-9. Left pupil dilated, nonreactive, right pupil sluggish & 3mm.”

## Slide 13
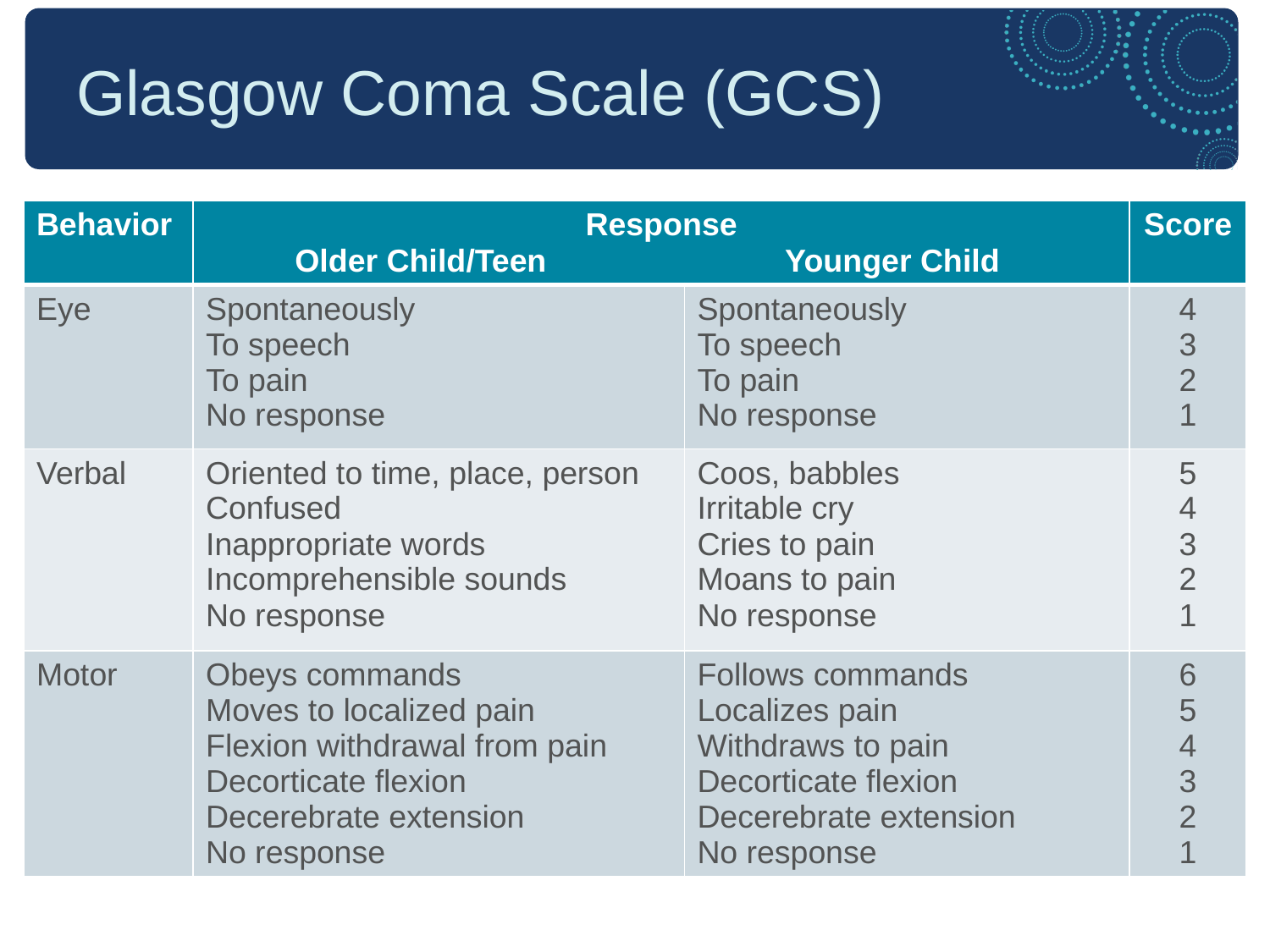

# Glasgow Coma Scale (GCS)
| Behavior | Response Older Child/Teen Younger Child | | Score |
| --- | --- | --- | --- |
| Eye | Spontaneously To speech To pain No response | Spontaneously To speech To pain No response | 4 3 2 1 |
| Verbal | Oriented to time, place, person Confused Inappropriate words Incomprehensible sounds No response | Coos, babbles Irritable cry Cries to pain Moans to pain No response | 5 4 3 2 1 |
| Motor | Obeys commands Moves to localized pain Flexion withdrawal from pain Decorticate flexion Decerebrate extension No response | Follows commands Localizes pain Withdraws to pain Decorticate flexion Decerebrate extension No response | 6 5 4 3 2 1 |

## Slide 14
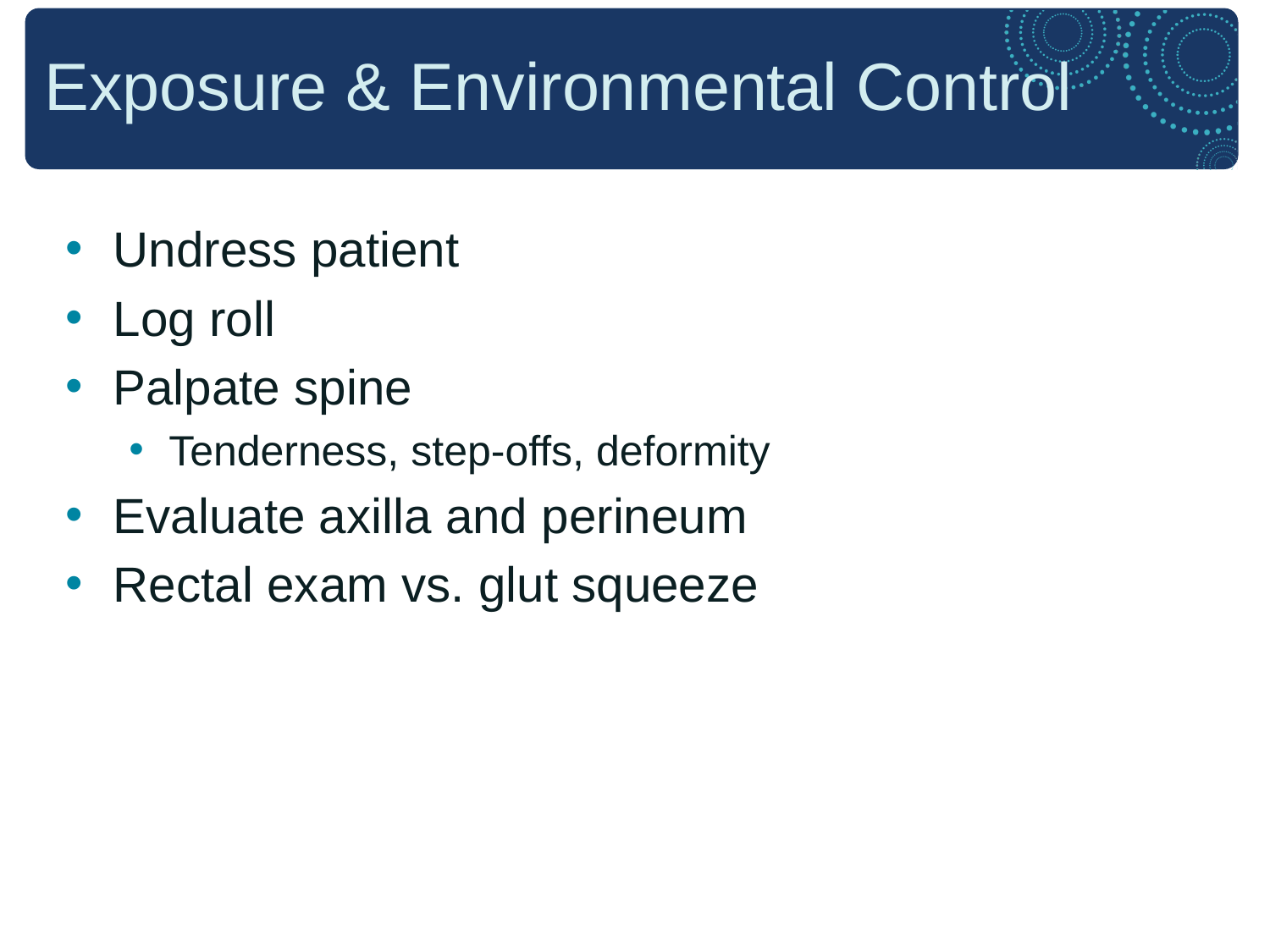

# Exposure & Environmental Control
Undress patient
Log roll
Palpate spine
Tenderness, step-offs, deformity
Evaluate axilla and perineum
Rectal exam vs. glut squeeze

## Slide 15
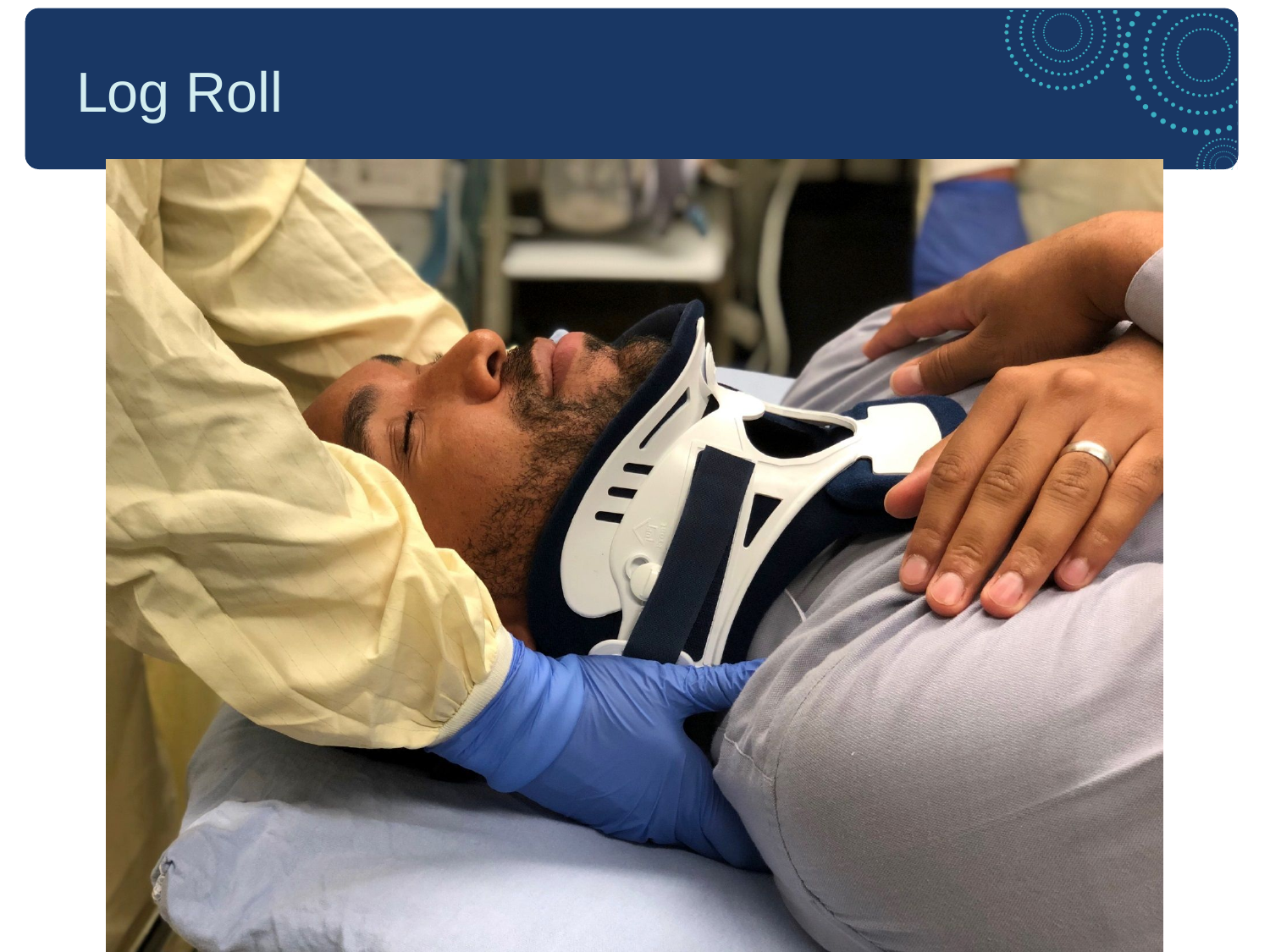

# Log Roll

## Slide 16
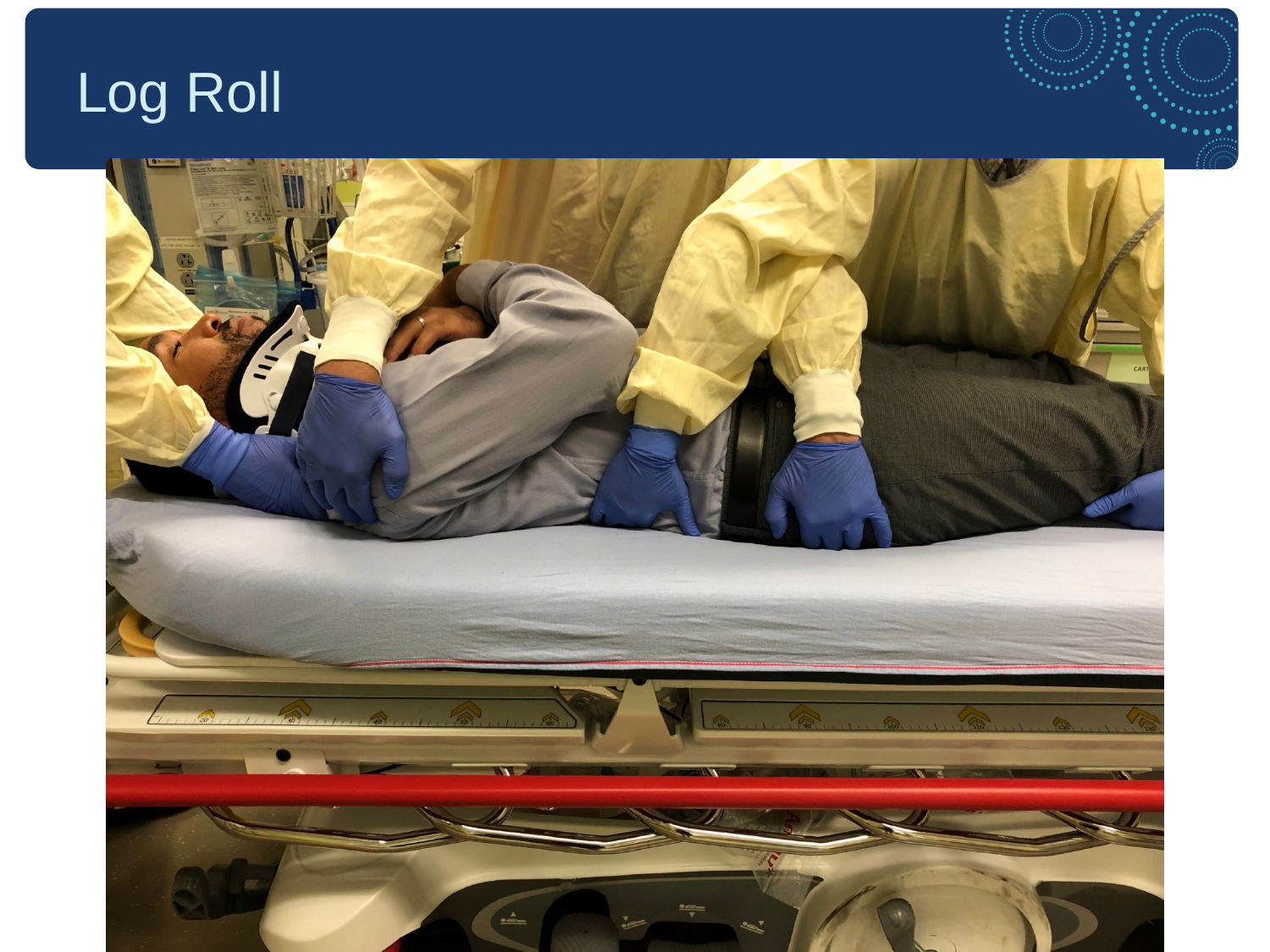

# Log Roll

## Slide 17
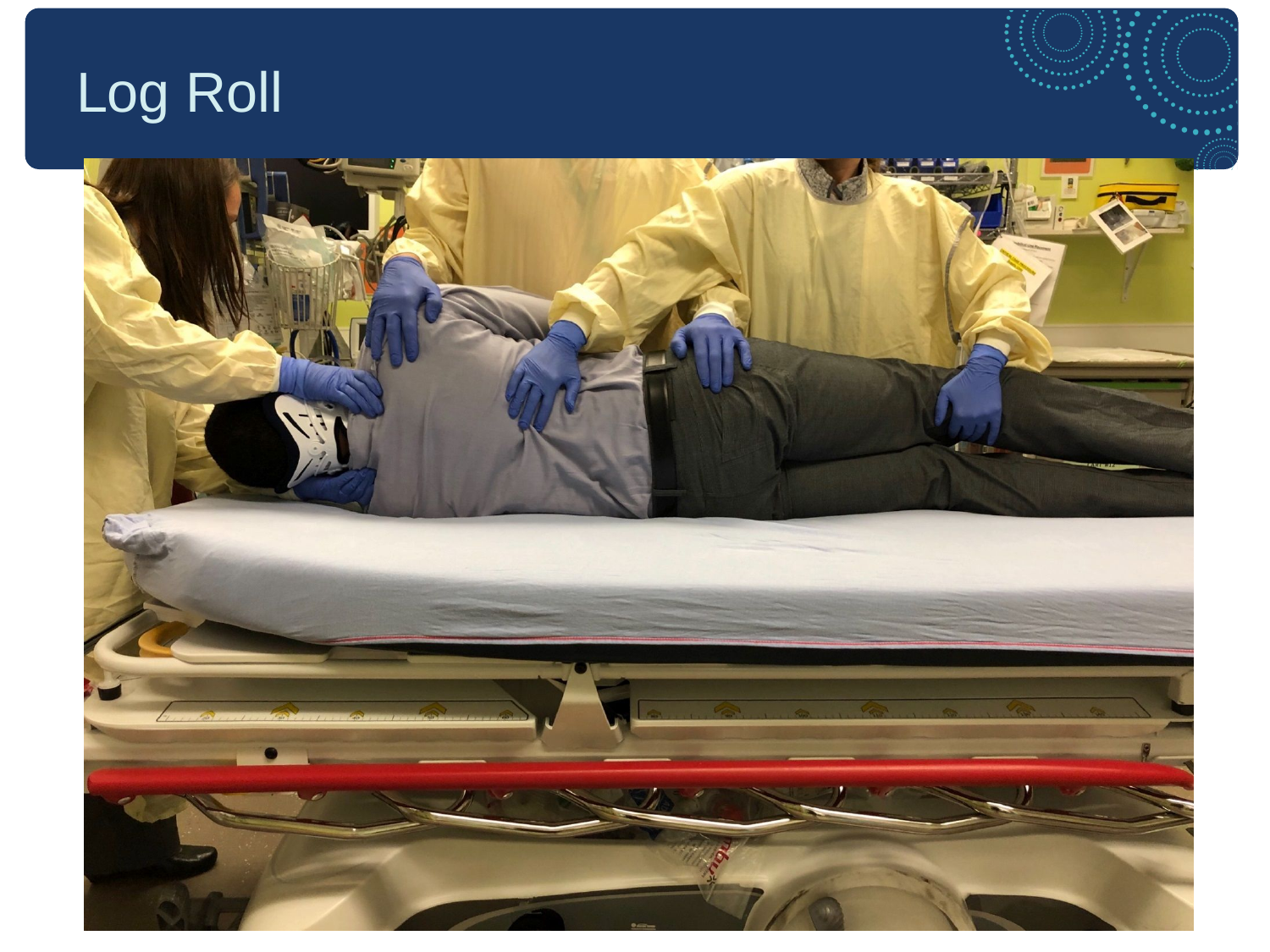

# Log Roll

## Slide 18
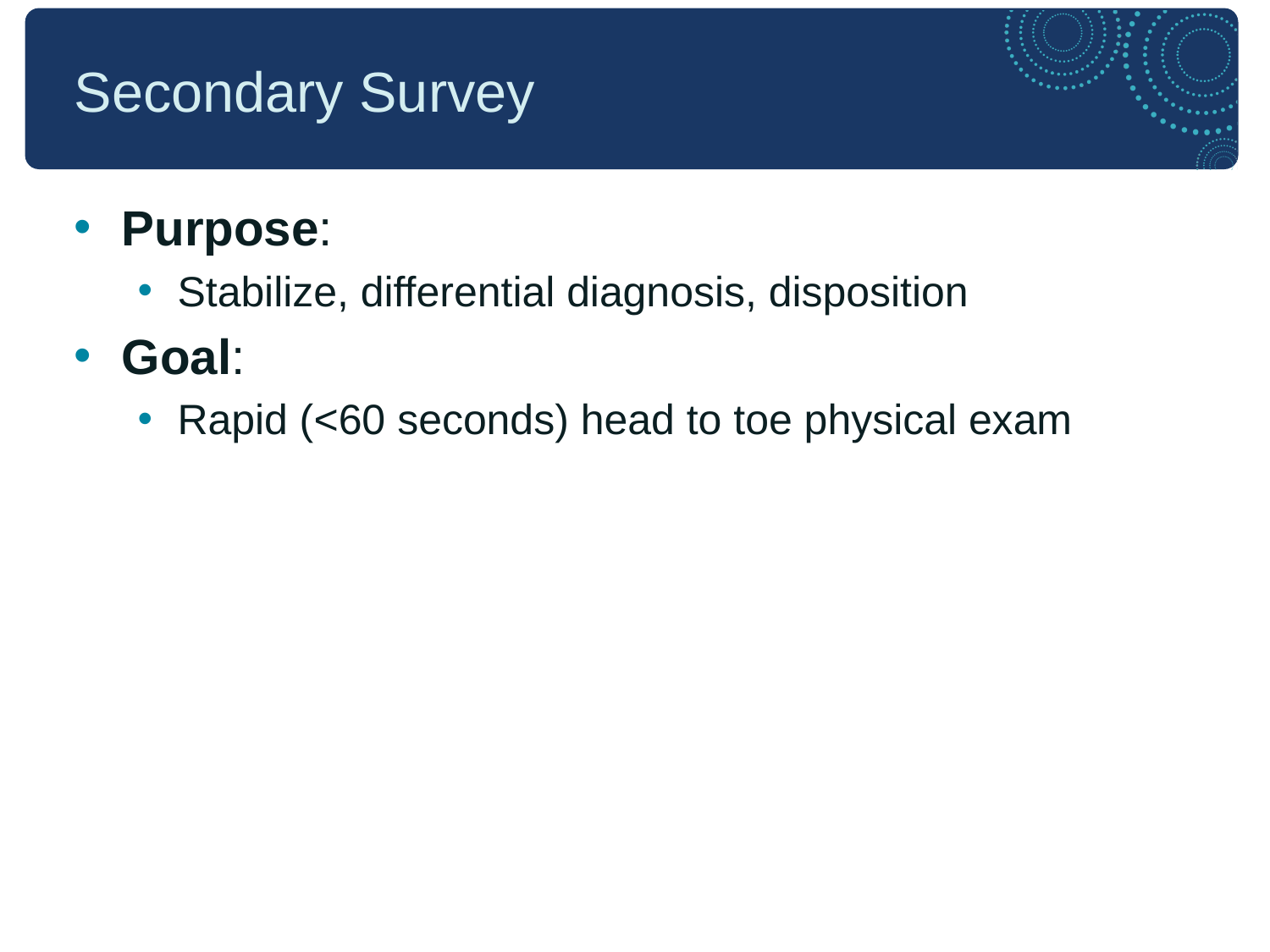

# Secondary Survey
Purpose:
Stabilize, differential diagnosis, disposition
Goal:
Rapid (<60 seconds) head to toe physical exam

## Slide 19
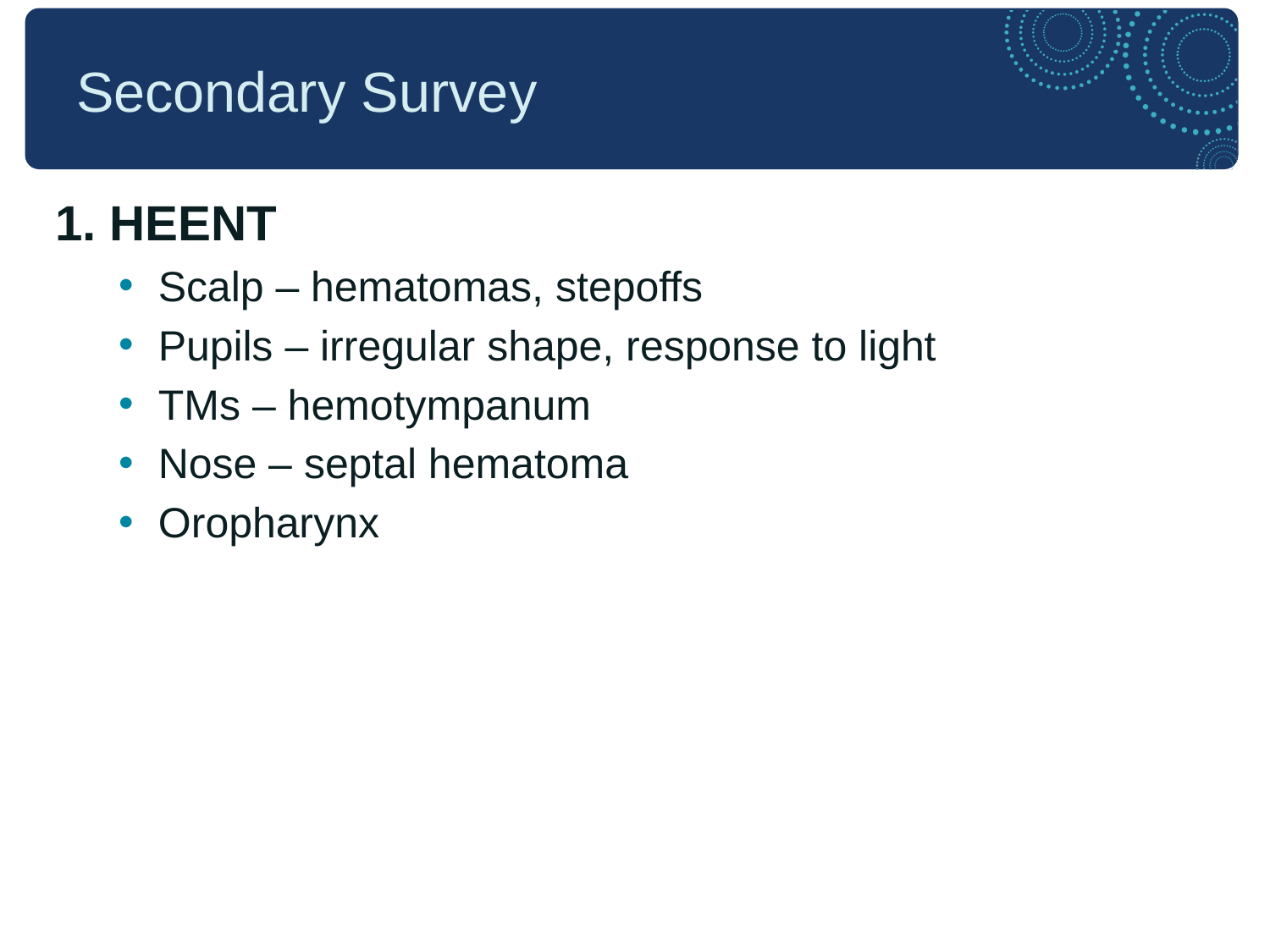

# Secondary Survey
1. HEENT
Scalp – hematomas, stepoffs
Pupils – irregular shape, response to light
TMs – hemotympanum
Nose – septal hematoma
Oropharynx

## Slide 20
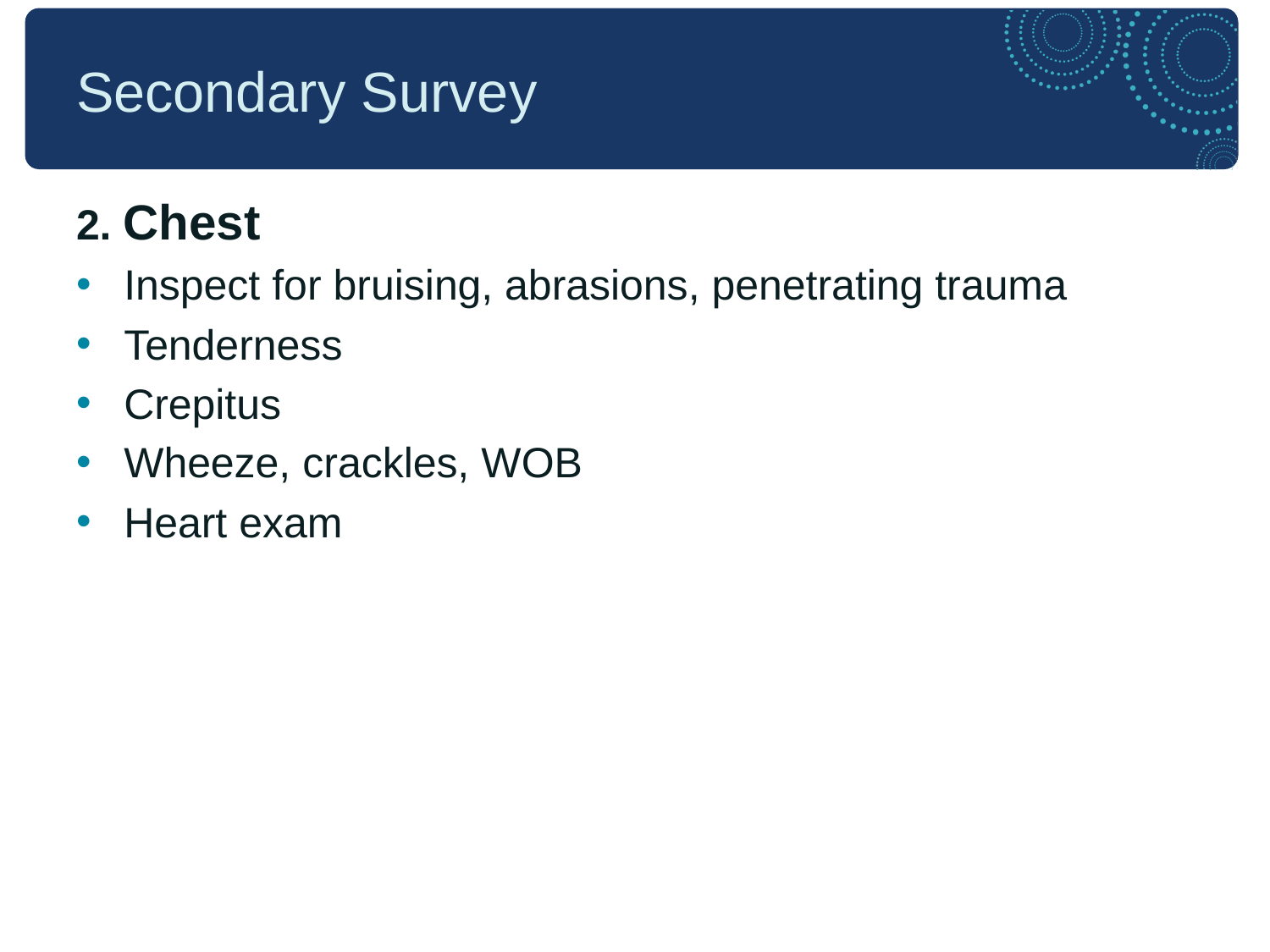

# Secondary Survey
2. Chest
Inspect for bruising, abrasions, penetrating trauma
Tenderness
Crepitus
Wheeze, crackles, WOB
Heart exam

## Slide 21
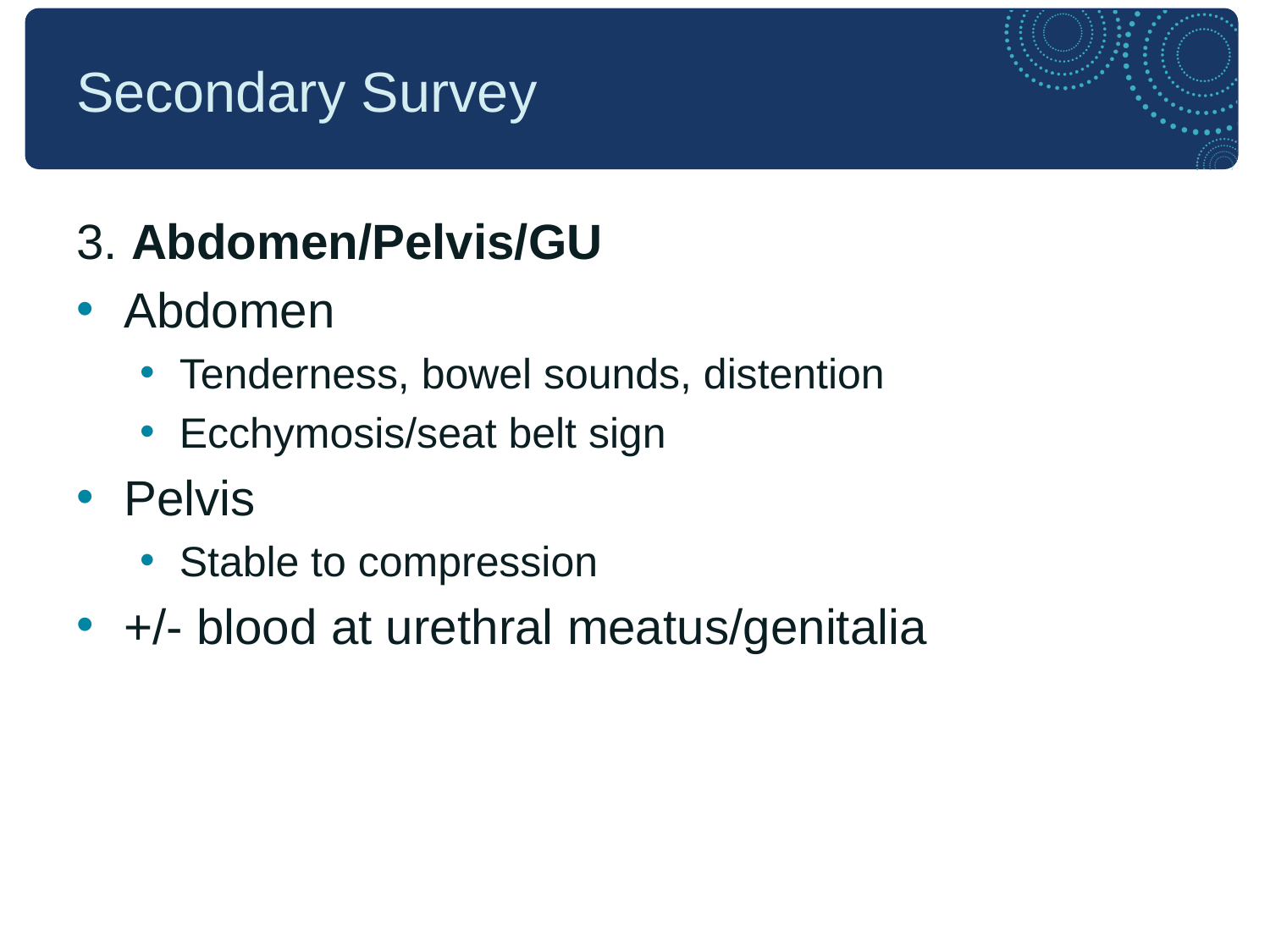

# Secondary Survey
3. Abdomen/Pelvis/GU
Abdomen
Tenderness, bowel sounds, distention
Ecchymosis/seat belt sign
Pelvis
Stable to compression
+/- blood at urethral meatus/genitalia

## Slide 22
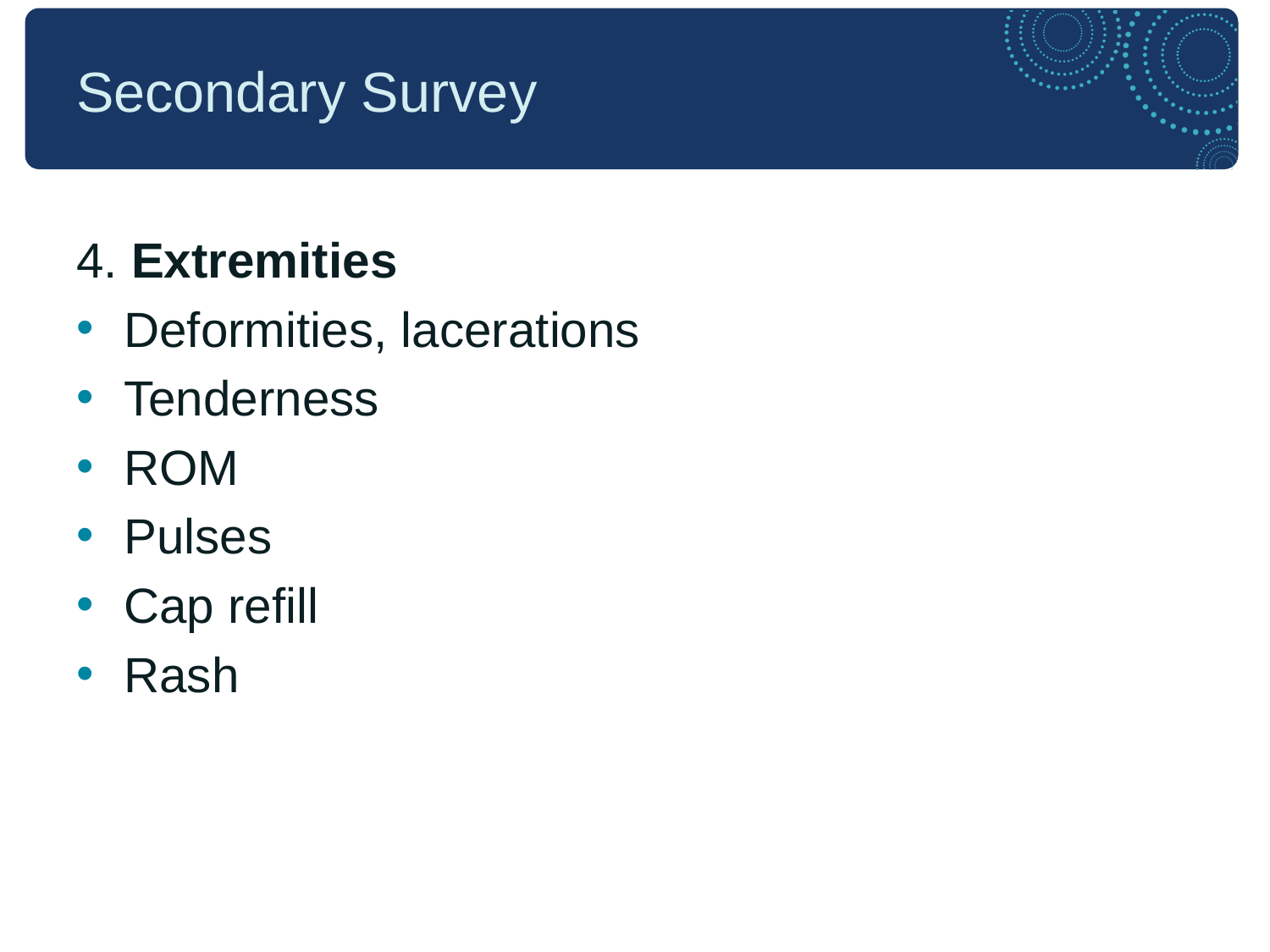

# Secondary Survey
4. Extremities
Deformities, lacerations
Tenderness
ROM
Pulses
Cap refill
Rash

## Slide 23
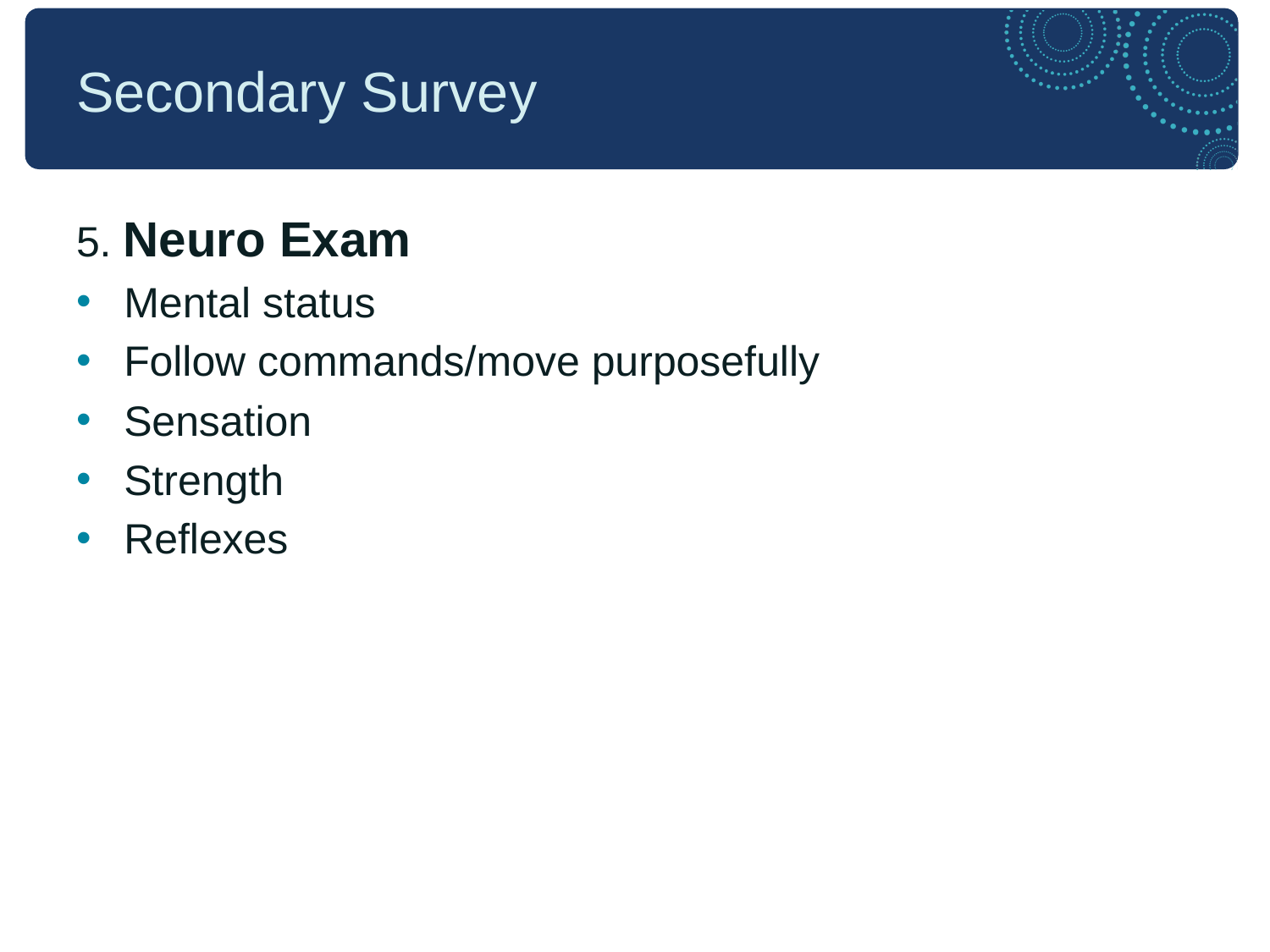

# Secondary Survey
5. Neuro Exam
Mental status
Follow commands/move purposefully
Sensation
Strength
Reflexes

## Slide 24
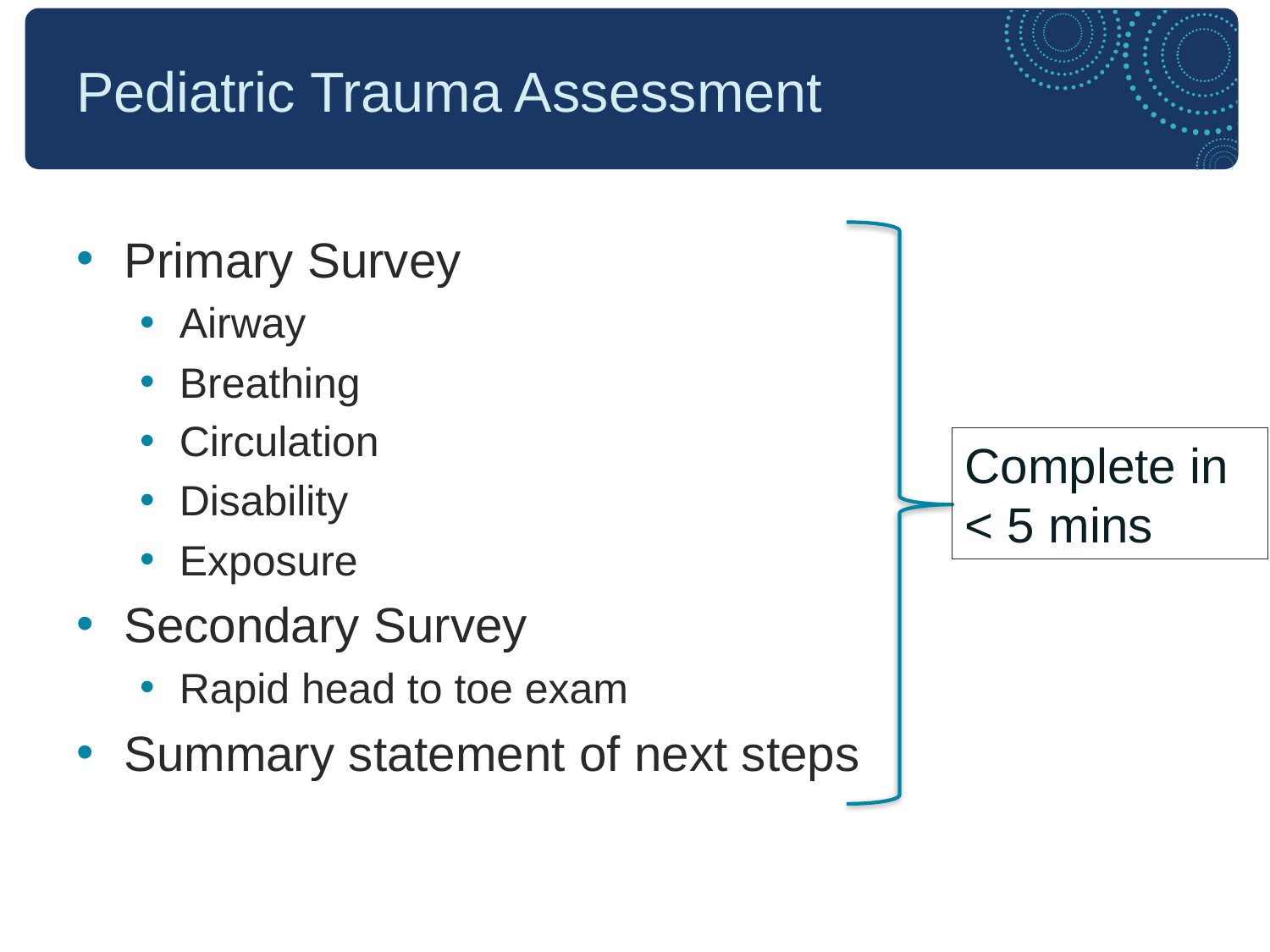

# Pediatric Trauma Assessment
Primary Survey
Airway
Breathing
Circulation
Disability
Exposure
Secondary Survey
Rapid head to toe exam
Summary statement of next steps
Complete in
< 5 mins

## Slide 25
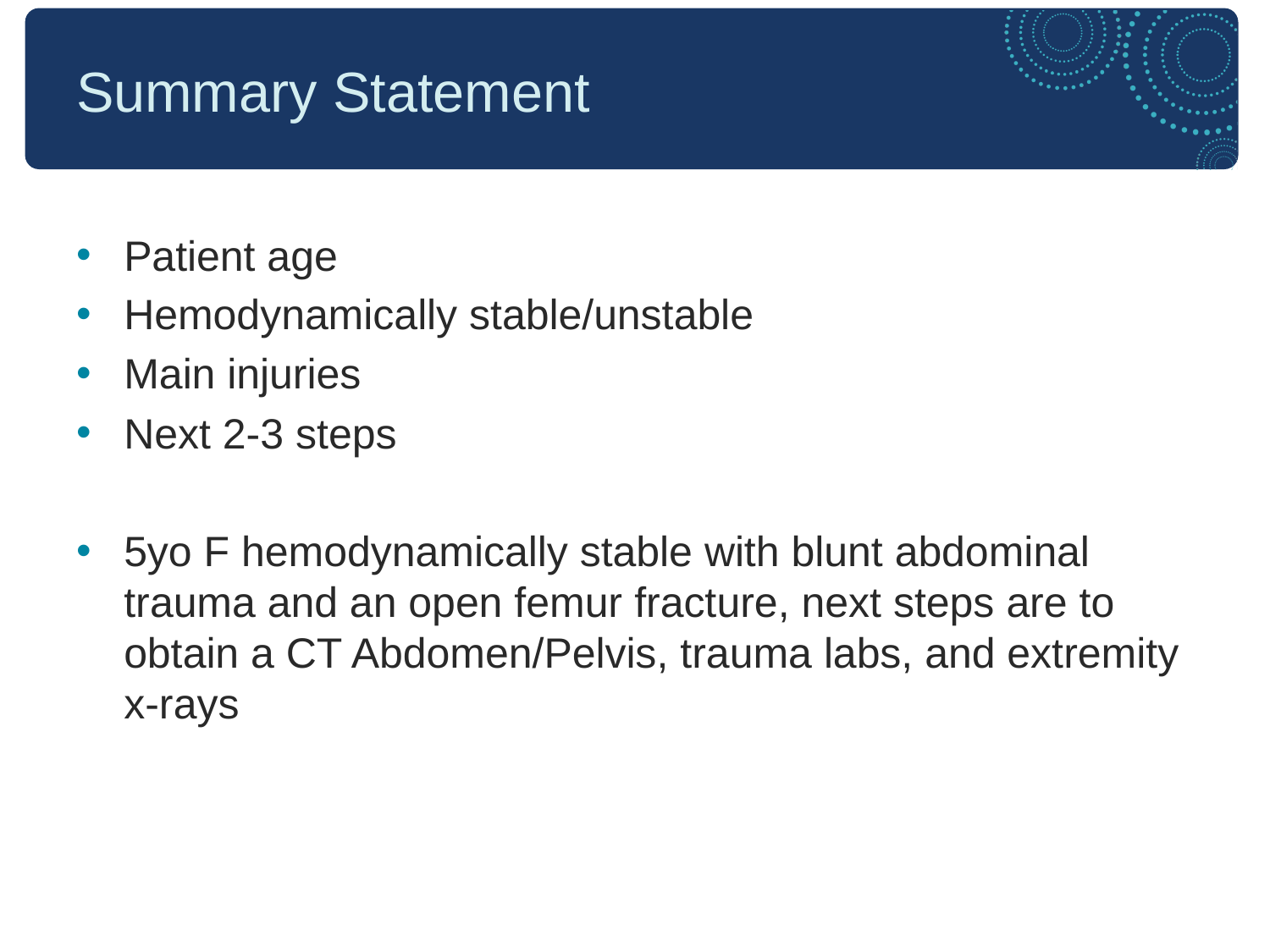

# Summary Statement
Patient age
Hemodynamically stable/unstable
Main injuries
Next 2-3 steps
5yo F hemodynamically stable with blunt abdominal trauma and an open femur fracture, next steps are to obtain a CT Abdomen/Pelvis, trauma labs, and extremity x-rays

## Slide 26
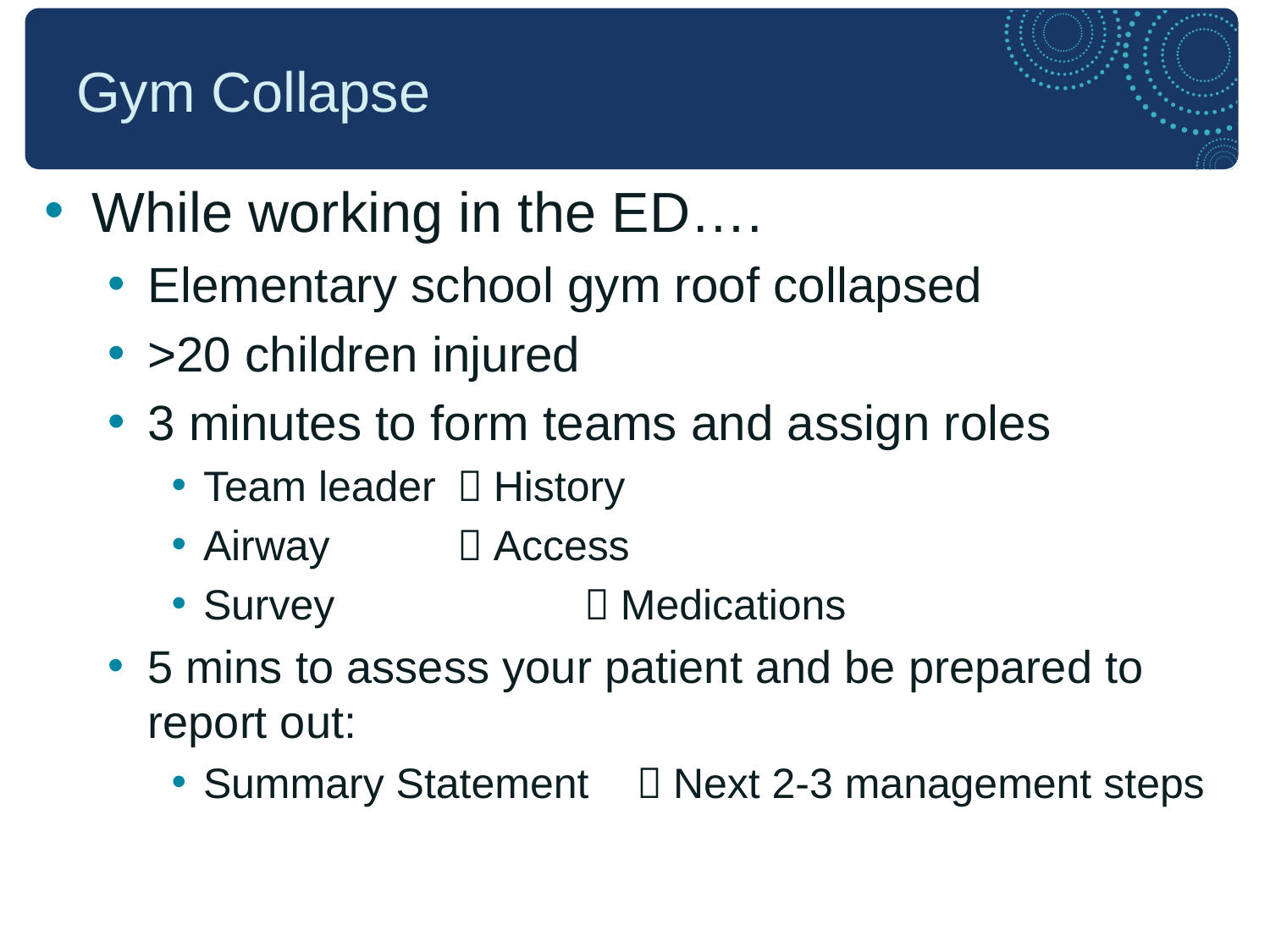

# Gym Collapse
While working in the ED….
Elementary school gym roof collapsed
>20 children injured
3 minutes to form teams and assign roles
Team leader	 History
Airway		 Access
Survey		 Medications
5 mins to assess your patient and be prepared to report out:
Summary Statement  Next 2-3 management steps
