# Supplementary material for: Pediatric Emergency Medicine Disaster Simulation Curriculum: The 5-Minute Trauma Assessment for Pediatric Residents (TRAP-5)
Source: MedEdPORTAL. 2020 Aug 21;16:10940. doi: 10.15766/mep_2374-8265.10940 (PMC7449578; doi:10.15766/mep_2374-8265.10940)
Supplement: Supplementary file 1 — Simulation Case Template.docxSimulation Environmental Preparation.docxSimulation Images and Materials.pptxCommunication Tools.docxDebriefing Materials.docxDidactic PowerPoint Presentation.pptxEvaluation Form.docxCritical Actions Checklist.docx [file mep_2374-8265.10940-s001.zip › G. Evaluation Form.docx]

**Appendix G: Pediatric Disaster Simulation Evaluation Form**

**Instructor(s):** **Date:**

**Case Presented:** Pediatric Trauma Assessment

|  | Strongly Disagree | Disagree | Neutral | Agree | Strongly Agree |
| --- | --- | --- | --- | --- | --- |
| The case presented during the simulation was relevant to my work | 1 | 2 | 3 | 4 | 5 |
| The simulation case was realistic | 1 | 2 | 3 | 4 | 5 |
| The simulation case was effective in teaching basic resuscitation skills | 1 | 2 | 3 | 4 | 5 |
| The debrief promoted reflection and team discussion | 1 | 2 | 3 | 4 | 5 |
| The group discussion helped me develop and prioritize evaluation and management options for a child with acute trauma | 1 | 2 | 3 | 4 | 5 |
| The facilitators created a safe environment for discussion and exploration | 1 | 2 | 3 | 4 | 5 |

After participating in this session how confident are you in your ability to:

|  | Very Unconfident | Unconfident | Neutral | Confident | Very Confident |
| --- | --- | --- | --- | --- | --- |
| Demonstrate ability to assess and emergently manage airway, breathing, and circulation | 1 | 2 | 3 | 4 | 5 |
| Apply concepts to control external bleeding via direct pressure and tourniquet application | 1 | 2 | 3 | 4 | 5 |
| Formulate a list of possible diagnoses and prioritize elements of evaluation | 1 | 2 | 3 | 4 | 5 |
| Obtain initial elements of trauma evaluation including imaging, labs, and specialty consultation | 1 | 2 | 3 | 4 | 5 |
| Utilize effective communication skills during a trauma resuscitation | 1 | 2 | 3 | 4 | 5 |
| Construct a disposition plan after stabilization in the emergency department | 1 | 2 | 3 | 4 | 5 |

Describe one or more ways this session will change how you do your job.

How could we improve this simulation and workshop?

Additional Comments:
