# Supplementary material for: Pediatric Emergency Medicine Disaster Simulation Curriculum: The 5-Minute Trauma Assessment for Pediatric Residents (TRAP-5)
Source: MedEdPORTAL. 2020 Aug 21;16:10940. doi: 10.15766/mep_2374-8265.10940 (PMC7449578; doi:10.15766/mep_2374-8265.10940)
Supplement: Supplementary file 1 — Simulation Case Template.docxSimulation Environmental Preparation.docxSimulation Images and Materials.pptxCommunication Tools.docxDebriefing Materials.docxDidactic PowerPoint Presentation.pptxEvaluation Form.docxCritical Actions Checklist.docx [file mep_2374-8265.10940-s001.zip › H. Critical Actions Checklist.docx]

**Appendix H: Pediatric Disaster Simulation Critical Action Checklist**

**Critical Action 1 – Completion of primary survey**

Primary survey completed within 2 minutes

Primary survey completed in appropriate order (ABCDE)

Clothing removal of patient

Log-rolling patient

Primary survey called out in a loud voice for all team members to hear

Life-threatening abnormalities addressed prior to continuing primary survey

**Critical Action 2 – Completion of assessment and determination of next priorities**

Secondary survey completed after primary survey

Giving summary statement and stating next priorities within 5 minutes

- Priorities may include: obtaining IV access, IV fluid or blood product administration, cervical spine immobilization, obtaining imaging/labs, surgical consultation

**Critical Action 3 – Utilize clear communication and maintain team roles**

Team roles assigned at the beginning of the scenario and roles maintained throughout the simulation

High-quality clear communication techniques consistently used throughout the simulation including closed-loop communication, directed call-out, and summary statements
